# Mechanosensitive snoRNA-like circular RNA sno-circCNOT1 drives endothelial dysfunction and atherosclerosis

**DOI:** 10.7150/thno.122995

**Published:** 2026-01-01

**Authors:** Lianru Bi, Yihao Zhu, Ziqi Chen, Yiying Yang, Yanlong Leng, Huijie Wang, Jiajie Pan, Xiaozhe Zhang, Zekai Zeng, Yunjun Liang, Guifu Wu, Wendong Fan

**Affiliations:** 1 Department of Cardiology, the Eighth Affiliated Hospital of Sun Yat-sen University, Shenzhen, 518033, Guangdong, People's Republic of China.; 2 Department of Cardiology, the First Affiliated Hospital of Sun Yat-sen University, Guangzhou, 510080, Guangdong, People's Republic of China.; 3 NHC Key Laboratory of Assisted Circulation and Vascular Diseases, Sun Yat-sen University, Guangzhou, 510080, Guangdong, People's Republic of China.; 4 Guangdong Innovative Engineering and Technology Research Center for Assisted Circulation, Shenzhen, Guangdong, People's Republic of China.; 5 MOE Key Laboratory of Laser Life Science & Guangdong Provincial Key Laboratory of Life Science, College of Biophotonics, School of Optoelectronic Science and Engineering, South China Normal University, Guangzhou, 510631, Guangdong, People's Republic of China.

**Keywords:** shear stress, atherosclerosis, circRNAs, pyroptosis, inflammation

## Abstract

**Rationale:** Hemodynamic shear stress critically influences atherosclerosis progression, yet the molecular mechanisms linking biomechanical stimuli to endothelial activation and vascular pathology remain poorly understood. While circular RNAs (circRNAs) participate in endothelial mechanotransduction, the role of mechanosensitive small nucleolar RNA (snoRNA)-like circRNA—a unique subclass harboring snoRNA sequences—in atherosclerosis is unexplored.

**Methods:** We characterized sno-circCNOT1 using high-throughput RNA sequencing, RNA interference, immunofluorescence, and co-immunoprecipitation. Functional studies were performed in endothelial cells and *ApoE⁻/⁻* mice to assess its role in pyroptosis and atherogenesis. Mechanistic investigations included RNA pull-down, mass spectrometry, and gain- and loss-of-function assays to identify sno-circCNOT1-interacting proteins and downstream signaling.

**Results:** We identified sno-circCNOT1, a circular RNA derived from *CNOT1* exon 17 and intron 17, which incorporates snoRNA SNORA50A. Its expression was upregulated by pro-atherogenic interleukin-1β and pathological oscillatory shear stress, but downregulated by laminar shear stress. Functionally, sno-circCNOT1 mediated shear stress-dependent regulation of endothelial pyroptosis and inflammation. Endothelial-specific overexpression of sno-circCNOT1 aggravated atherosclerotic lesion formation in *ApoE⁻/⁻* mice. Mechanistically, its snoRNA-like motif was essential for nuclear localization and function. sno-circCNOT1 bound the IF-ROD domain of lamin A/C (LMNA), stabilizing LMNA and facilitating its interaction with the N-terminal domain of methyltransferase-like 14 (METTL14-N), thereby enhancing METTL14 stability. This axis activated NOD-like receptor protein 3 (NLRP3) and amplified endothelial inflammation. Conversely, overexpression of METTL14-N to disrupt this signaling axis attenuates endothelial dysfunction and atherosclerosis progression.

**Conclusions:** sno-circCNOT1 is a mechanosensitive snoRNA-like circRNA that promotes endothelial pyroptosis and atherogenesis via the LMNA/METTL14/NLRP3 axis. METTL14-N offers a protein-based therapeutic approach, positioning this regulatory pathway as a druggable target for atherosclerosis.

## Introduction

Atherosclerosis, characterized as a chronic inflammatory process arising from endothelial dysfunction and vascular injury, is tightly regulated by hemodynamic shear stress-the frictional force generated by blood flow acting upon the vascular endothelium 1,2. Physiological laminar shear stress (LSS) produced by unidirectional blood flow in straight vessels maintains endothelial homeostasis and vascular integrity, whereas pathological low and oscillatory shear stress (OSS) generated by disturbed flow at arterial curvatures or bifurcations promotes endothelial activation and disease progression 3. Under atheroprone flow, endothelial cells (ECs) undergo pyroptosis, an inflammatory form of programmed cell death driven by the NLRP3 inflammasome, along with gasdermin-D-mediated membrane pore formation and consequent release of proinflammatory cytokines including interleukin-1β (IL-1β) 4,5. This pyroptotic cascade intensifies vascular inflammation by increasing leukocyte adhesion and macrophage infiltration, thereby accelerating atherosclerotic plaque development.

Circular RNAs (circRNAs), covalently closed RNA molecules lacking both 5′ caps and 3′ poly(A) tails, exhibit enhanced structural stability compared to their linear counterparts 6,7. The evolutionary conservation and tissue-specific expression patterns suggest critical regulatory functions in diverse biological processes. Emerging evidence, including our own, has linked circRNAs to atherogenesis through mechanisms such as microRNA sponging and modulation of protein interactions 8-12. However, the functional relevance of mechanosensitive snoRNA-like circRNAs—a distinct subclass harboring embedded small nucleolar RNA (snoRNA) sequences—remains completely unexplored.

Here, we characterize sno-circCNOT1, a novel shear-responsive circRNA derived from exon 17 and intron 17 of the *CNOT1* gene, uniquely incorporating the intronic snoRNA SNORA50A. The expression of sno-circCNOT1 is upregulated by pro-atherogenic stimuli such as IL-1β and OSS, but suppressed by atheroprotective LSS. Functionally, endothelial sno-circCNOT1 mediates shear stress-dependent regulation of pyroptosis and inflammation, thereby promoting atherosclerosis progression in *ApoE⁻/⁻* mice. Mechanistically, sno-circCNOT1 requires its snoRNA-like motif for nuclear localization, where it stabilizes lamin A/C (LMNA) and triggers a signaling cascade that activates METTL14 and NLRP3. Notably, targeting the sno-circCNOT1-LMNA-METTL14-NLRP3 axis attenuates atherosclerosis in *ApoE⁻/⁻* mice, highlighting its therapeutic potential. Collectively, these findings uncover an unexpected role for this snoRNA-containing circRNA as a key component of a novel mechanotransduction pathway that governs vascular inflammation and atherogenesis.

## Methods

Extended methods sections are available in the Supplemental Material.

### Animals

For sno-circCNOT1 overexpression, forty 6-week-old male *ApoE⁻/⁻* mice (Guangdong Pharmacokang, China) were randomly assigned to three groups: a blank control group (n = 10), an AAV-negative control (AAV-NC) group (n = 15), and an AAV-sno-circCNOT1 treatment group (n = 15). After two weeks on a normal diet, mice (excluding the blank control group) underwent partial left carotid artery ligation (PCL), followed by in situ injections of either AAV-NC (1×10¹⁰ VG; 50 μL) or AAV-sno-circCNOT1 (1×10¹⁰ VG; 50 μL) viral vectors (GeneChem Co., Ltd., Shanghai, China). Following AAV treatment, all mice received a high-fat diet (60% kcal from fat; medicience, MD12033) for 10 weeks, followed by a modified high-fat diet (41% kcal fat, 0.15% w/w cholesterol; medicience, MD12015) for 4 weeks to induce atherosclerosis. At the endpoint, mice were euthanized, and whole blood, carotid arteries, and aorta specimens were collected for further analysis.

For Mettl14-N overexpression, forty 8-week-old male *ApoE⁻/⁻* mice (Guangdong Pharmackang, China) were randomly assigned into three groups: a blank group (n = 10), an AAV-FLAG-GFP control group (n = 15), and an AAV-FLAG-Mettl14-N treatment group (n = 15). After three weeks on a normal diet, mice (excluding the blank control group) were injected with either AAV-FLAG-GFP (1×10¹⁰ VG; 200 μL) or AAV-FLAG-Mettl14-N (1×10¹⁰ VG; 200 μL) viral vectors (GeneChem Co., Ltd., Shanghai, China). Three weeks post-injection, PCL was performed on the left carotid artery. The mice were subsequently maintained on a high-fat diet (41% kcal fat, 0.15% cholesterol; medicience, MD12015) for 12 weeks to establish an atherosclerotic model. At the study endpoint, mice were sacrificed, and whole blood, bilateral carotid arteries, and aortic tissues were harvested for downstream analysis.

All experimental mice were maintained in a specific pathogen-free environment at 21 °C ± 2 °C, with unrestricted access to standard chow and water. All animal procedures were reviewed and approved by the Animal Care and Use Committee of Sun Yat-Sen University (approval numbers: SYSU-IACUC-2023-001588 and SYSU-IACUC-2024-002861).

### Statistical analysis

Data are expressed as the mean ± standard error of the mean (SEM) from at least three independent experiments. Statistical comparisons between two groups were analyzed using an unpaired two-tailed Student's *t* test. Multiple group comparisons were conducted using one-way or two-way analysis of variance (ANOVA) followed by Tukey's post hoc test. Statistical evaluations were performed in GraphPad Prism 9.5 (GraphPad Software), and *P* < 0.05 was considered statistically significant.

## Results

### Endothelial sno-circCNOT1 is upregulated by pro-atherosclerotic IL-1β but downregulated by anti-atherosclerotic LSS

**Figure 1:**
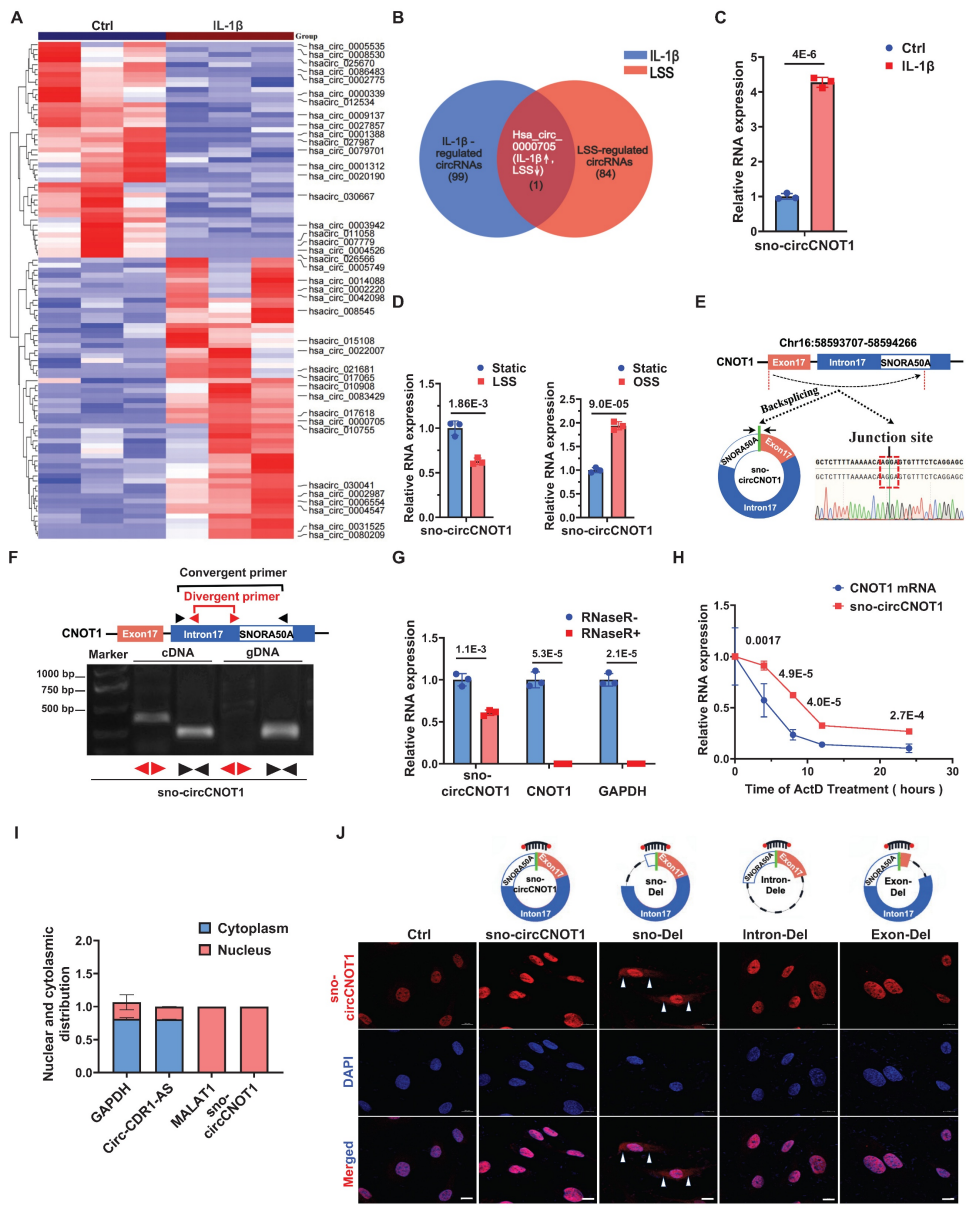
** Endothelial sno-circCNOT1 is upregulated by pro-atherosclerotic interleukin-1β (IL-1β) but downregulated by anti-atherosclerotic shear stress. A**, Heatmap of circRNA expression profiles in IL-1β-stimulated HUVECs (|log2(fold change) | > 1 and *p* < 0.05). Top 20 upregulated and downregulated circRNAs were shown. **B**, Venn diagram depicting the intersection of IL-1β-regulated circRNAs (blue circle; |Fold Change| > 2.0, P < 0.05; n = 99) and LSS-regulated circRNAs (|Fold Change| > 1.5, P < 0.05; n = 84). Hsa_circ_0000705 was the only circRNA significantly regulated by both stimuli (IL-1β↑, LSS↓). **C and D**, RT-qPCR quantification of sno-circCNOT1 levels in IL-1β-treated (**C**) or LSS/OSS-exposed (**D**) HUVECs, normalized to GAPDH (n = 3). **E,** Genomic structure of sno-circCNOT1 derived from the CNOT1 exon 17 and partial intron 17 (containing SNORA50A sequence). The back-splice junction of sno-circCNOT1 was validated by Sanger sequencing. **F,** sno-circCNOT1 can be amplified by divergent primers in cDNA (due to back-splicing) but not in genomic DNA, while convergent primers amplify linear transcripts in both cDNA and DNA. **G,** RNase R resistance assay demonstrating enhanced stability of sno-circCNOT1 versus linear CNOT1/GAPDH mRNAs (n = 3). **H,** RNA stability of sno-circCNOT1 and CNOT1 following actinomycin D treatment was assessed by RT-qPCR (n = 3). **I,** Subcellular fractionation confirming nuclear enrichment of sno-circCNOT1 in HUVECs (n = 3). Controls: MALAT1 (nuclear), circRNA-CDR1-AS1/GAPDH (cytoplasmic). **J,** Subcellular localization analysis of wild-type (WT) sno-circCNOT1 and its deletion mutants in HUVECs. Constructs include: snoRNA motif-deleted mutant (sno-Del), exon 17-deleted mutant (Exon-Del), and intron 17-deleted mutant (Intron-Del). Cy3: sno-circCNOT1 probes; DAPI: nuclei; VE-cadherin: green. The white arrows indicated the cytoplasmic localization of sno-Del. Scale bar: 10 μm. Statistical analyses were performed using an unpaired two-tailed Student's *t*-test. Data are presented as mean ± SEM. *P* values indicated. **E**, **F** and **J** were created by using Figdraw.

To investigate whether pro-atherogenic stimuli modulate endothelial circRNA profiles, we treated ECs with IL-1β and performed genome-wide circRNA sequencing (circRNA-seq). This analysis identified 99 significantly dysregulated circRNAs (|log₂ fold change| > 1; *P* < 0.05; 56 upregulated, 43 downregulated; Figure 1A), establishing an IL-1β-responsive circRNA signature. To identify circRNAs potentially orchestrating endothelial responses under both anti- and pro-atherogenic conditions, we took the intersection of this IL-1β-induced signature with our previous dataset of LSS-regulated circRNAs 10. This intersection analysis revealed hsa_circ_0000705 (here after designated sno-circCNOT1), which showed robust expression (Figure 1A) and exhibited convergent regulation—being suppressed by anti-atherogenic LSS while induced by pro-atherogenic IL-1β and OSS (Figure 1B through 1D). Consistently, sno-circCNOT1 was also regulated by other atherogenic and anti-atherogenic stimuli. TNFα and ox-LDL markedly increased its expression, whereas the anti-atherogenic drug atorvastatin reduced it (Figure S1). Moreover, in human aortic endothelial cells (HAECs), sno-circCNOT1 was similarly upregulated by IL-1β and OSS, but suppressed under LSS (Figure S2), confirming that its regulation pattern is conserved across endothelial contexts. Therefore, we selected this circRNA for further functional characterization.

Interestingly, hsa_circ_0000705 is a snoRNA-like circRNA originating from exon 17 and a portion of intron 17 of the *CNOT1* gene, which harbors the H/ACA box snoRNA, SNORA50A, within intron 17 (Figure 1E). Therefore, we designated this circRNA as sno-circCNOT1. Notably, sno-circCNOT1 lacks the 5'-guACAuuu-3' sequence that containing the ACA motif located at the 3' end of mature SNORA50A. Using divergent primers flanking the back-splicing junction and Sanger sequencing, we validated that the junction sequence of sno-circCNOT1 matched the annotation in the circBase database (Figure 1E). Furthermore, sno-circCNOT1 was amplified by divergent primers from cDNA but not from genomic DNA (Figure 1F). Its partial resistance to RNase R digestion (Figure 1G) and actinomycin D treatment (Figure 1H) supports its circular structure. Subcellular fractionation followed by RT-qPCR (Figure 1I) and RNA fluorescence *in situ* hybridization (FISH) (Figure 1J) revealed that sno-circCNOT1 is predominantly localized in the nucleus of ECs. To determine whether the snoRNA-like sequence is essential for nuclear retention, we constructed vectors expressing wild-type (WT) sno-circCNOT1 or mutants lacking the snoRNA motif (sno-Del), exon 17 (Exon-Del), or intron 17 (Intron-Del) (Figure 1J). All mutants retained the same back-splicing junction as the WT, allowing detection with the same probes. Upon overexpression in ECs, only the sno-Del mutant exhibited cytoplasmic localization, while nuclear signal (representing endogenous WT sno-circCNOT1) persisted (Figure 1J), indicating that the sno-circCNOT1 requires its snoRNA-like motif for nuclear accumulation. Collectively, these findings establish sno-circCNOT1 as a nuclear-enriched, snoRNA-like circRNA that is dynamically regulated by both pro- and anti-atherogenic stimuli.

### sno-circCNOT1 mediates the regulatory effect of shear stress on endothelial pyroptosis and inflammation

**Figure 2:**
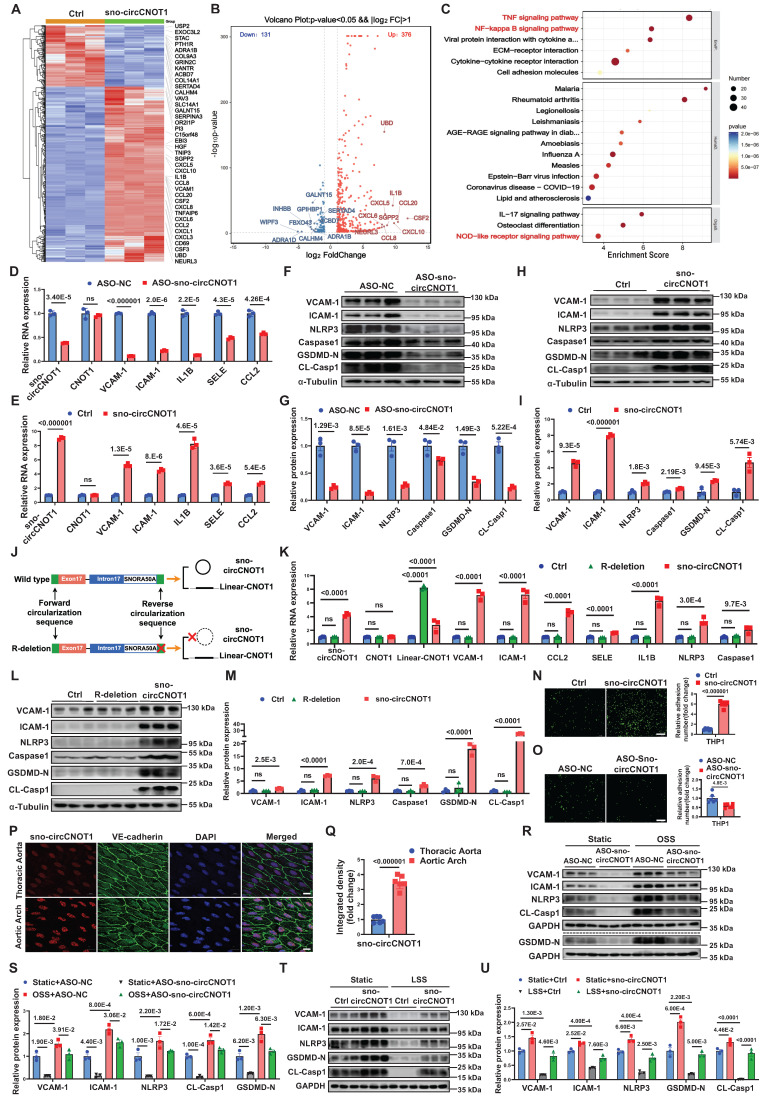
** sno-cirCNOT1 mediates the regulatory effect of shear stress on endothelial pyroptosis and inflammation. A,** Heatmap of differentially expressed genes (|log_2_FoldChange| > 1 and *p* < 0.05) in control (Ctrl) and sno-circCNOT1-overexpressing HUVECs. Labels indicate the top 20 upregulated and downregulated genes. **B**, Volcano plot of differentially expressed genes (|log_2_FoldChange| > 1 and *p* < 0.05). Labels indicate the top 10 upregulated and downregulated genes. **C**, KEGG enrichment of top 20 pathways regulated by sno-cirCNOT1. Dot color represents the *p*-value; dot size indicates enriched gene count. **D and E,** RT-qPCR analysis of sno-cirCNOT1, CNOT1, VCAM-1, ICAM-1, IL1B, SELE and CCL2 mRNA levels in sno-cirCNOT1-knockdown (**D**) or -overexpressing (**E**) HUVECs (normalized to GAPDH, n =3). **F through I,** Western blotting of inflammatory markers (VCAM-1 and ICAM-1) and pyroptosis markers (NLRP3, Caspase1, N-terminal GSDMD [GSDMD-N], and cleaved Caspase1 [CL-Casp1]) in sno-cirCNOT1-knockdown (**F and G**) or -overexpressing (**H and I**) HUVECs. **J,** Schematic of wild-type sno-cirCNOT1 vector and R-deletion mutant (lacking reverse circularization sequence; produces linear-CNOT1 transcript only). **K,** RT-qPCR analysis of sno-cirCNOT1, CNOT1, linear-CNOT1, VCAM-1, ICAM-1, CCL2, SELE, IL1B, NLRP3 and Caspase1 in HUVECs transduced with sno-cirCNOT1, R-deletion mutant or PLO5-ciR (control, Ctrl) lentivirus (normalized to GAPDH, n = 3). **L and M,** Western blotting of VCAM-1, ICAM-1, NLRP3, Caspase1, GSDMD-N and CL-Casp1 in HUVECs transduced with sno-circCNOT1, R-deletion or PLO5-ciR (Ctrl) lentivirus. **N and O,** Monocyte adhesion assays using fluorescently labeled THP-1 cells incubated with sno-cirCNOT1-knockdown (**N**) or -overexpressing (**O**) HUVECs. Scale bar: 200 μm. n = 6. **P and Q,** FISH assays showing elevated endothelial sno-circCNOT1 expression (red) in the mouse aortic arch versus thoracic aorta. Endothelial cells were stained with Cy3-labeled sno-cirCNOT1 probes (red), VE-cadherin antibody (green) and DAPI (blue). Scale bar: 10 μm. (**Q**) Quantification of the sno-cirCNOT1 expression (n = 6). **R and S,** HUVECs were transfected with ASO-NC (Ctrl) or ASO-sno-circCNOT1 for 24 hours, followed by exposure to oscillatory shear stress (OSS) (0.5 ± 4 dyne/cm^2^ at 1 Hz) for an additional 24 hours. Protein levels of VCAM-1, ICAM-1, NLRP3, GSDMD-N and CL-Casp1 were analyzed by western blotting (n = 3). **T and U,** Western blot analysis (T) and quantification (U) of indicated proteins in HUVECs transduced with control (Ctrl) or sno-circCNOT1 lentivirus for 48 hours, followed by exposure to atheroprotective LSS (15 dyne/cm^2^, 24 h). n = 3. Statistical analyses were performed using an unpaired two-tailed Student's t-test (**D** to **I** and **N** to **Q**) or one-way analysis of variance (ANOVA) with Tukey's post hoc test (**K** to **M)** or two-way analysis of variance (ANOVA) (**R** to **U**). Data are presented as mean ± SEM. *P* values indicated. **J** was created by using Figdraw.

We next investigated the functional role of sno-circCNOT1 in ECs. RNA sequencing (RNA-seq) of ECs following sno-circCNOT1 overexpression identified 507 differentially expressed genes (DEGs) (*P* < 0.05, |log_2_(FC)| > 1; Figure 2A and 2B). Kyoto Encyclopedia of Genes and Genomes (KEGG) pathway enrichment analysis indicated that the DEGs were mainly associated with pyroptosis and inflammation pathways, such as tumor necrosis factor α (TNF-α), NF-kappa B (NF-κB), and NOD-like receptor signaling pathways (Figure 2C). These findings suggest a potential role for sno-circCNOT1 in regulating endothelial pyroptosis and inflammation.

To test this hypothesis, we employed an antisense oligonucleotide (ASO)-based approach designed against the back-splicing junction of this circRNA to achieve selective knockdown of sno-circCNOT1 in ECs, thus minimizing nonspecific knockdown of the linear CNOT1 mRNA. RT-qPCR confirmed that sno-circCNOT1 knockdown reduced the expression of sno-circCNOT1, VCAM-1, ICAM-1, IL1B, SELE and CCL2, without affecting CNOT1 mRNA levels (Figure 2D), indicating CNOT1-independent regulation. Conversely, lentiviral overexpression of sno-circCNOT1 upregulated these pyroptotic and inflammatory markers while CNOT1 expression remained unchanged (Figure 2E). Western blot analysis further demonstrated that sno-circCNOT1 silencing suppressed (Figures 2F and 2G), while its overexpression enhanced (Figure 2H and 2I), the expression of pyroptotic and inflammatory markers, including VCAM-1, ICAM-1, NLRP3, Caspase-1, N-terminal cleavage fragment of GSDMD (GSDMD-N), and cleaved caspase-1 (CL-Casp1). Given the 82% genomic identity between human and mouse sno-circCNOT1 loci (Figure S3), we overexpressed human sno-circCNOT1 in mouse aortic endothelial cells (MAECs) to recapitulated key pathological phenotypes observed in human endothelial cells. Consistent phenotypes were subsequently observed in these cells (Figure S4).

Since not all transcripts generated by sno-circCNOT1 overexpression constructs are circularized into mature sno-circCNOT1, a small proportion of linear transcripts are inevitably produced during overexpression. To distinguish the functional effects of sno-circCNOT1 from those of its linear counterpart (linear-CNOT1), we designed a plasmid that exclusively expresses linear-CNOT1 by deleting the reverse circularization sequence (R-deletion) (Figure 2J). RT-qPCR analysis confirmed that this R deletion led to the exclusive production of the linear transcript, with no detectable overexpression of sno-circCNOT1 (Figure 2K). Consistent with expectations, the R-deletion group showed no notable difference from the control group (Figure 2L and 2M), supporting the conclusion that sno-circCNOT1 itself, rather than its linear counterpart, plays a key role in regulating endothelial pyroptosis and inflammation. Furthermore, we excluded the possibility that sno-circCNOT1 exerts its regulatory effects through SNORA50A, as overexpression of SNORA50A showed no impact on endothelial pyroptosis or inflammation (Figure S5). Additionally, THP-1 cell adhesion assays demonstrated that sno-circCNOT1 overexpression significantly enhanced monocyte adhesion (Figure 2N), while its knockdown markedly reduced adhesion (Figure 2O). Collectively, these findings highlight sno-circCNOT1 as a key regulator of endothelial pyroptosis and inflammation.

To further investigate the regulation of sno-circCNOT1 under shear stress *in vivo*, we evaluated its expression in mouse aorta using En face immunostaining combined with FISH. As expected, sno-circCNOT1 expression was elevated in the lesser curvature of the aortic arch—an area exposed to disturbed flow—compared to the thoracic aorta, which is characterized by steady laminar flow (Figure 2P and 2Q). To explore the functional role of sno-circCNOT1 in shear stress-induced responses, we silenced its expression in ECs and subjected the cells to OSS to determine whether sno-circCNOT1 knockdown could mitigate the pro-pyroptotic and pro-inflammatory effects of OSS. As anticipated, sno-circCNOT1 knockdown significantly attenuated OSS-induced upregulation of pyroptotic and inflammatory markers in ECs (Figure 2R and 2S; Figure S6). Similarly, sno-circCNOT1 overexpression partially reversed the anti-inflammatory and anti-pyroptotic effects induced by LSS (Figure 2T and 2U). Together, these findings suggest that sno-circCNOT1 regulates flow-dependent pyroptosis and inflammatory responses.

### sno-circCNOT1 interacts with LMNA protein and promotes its stabilization

**Figure 3:**
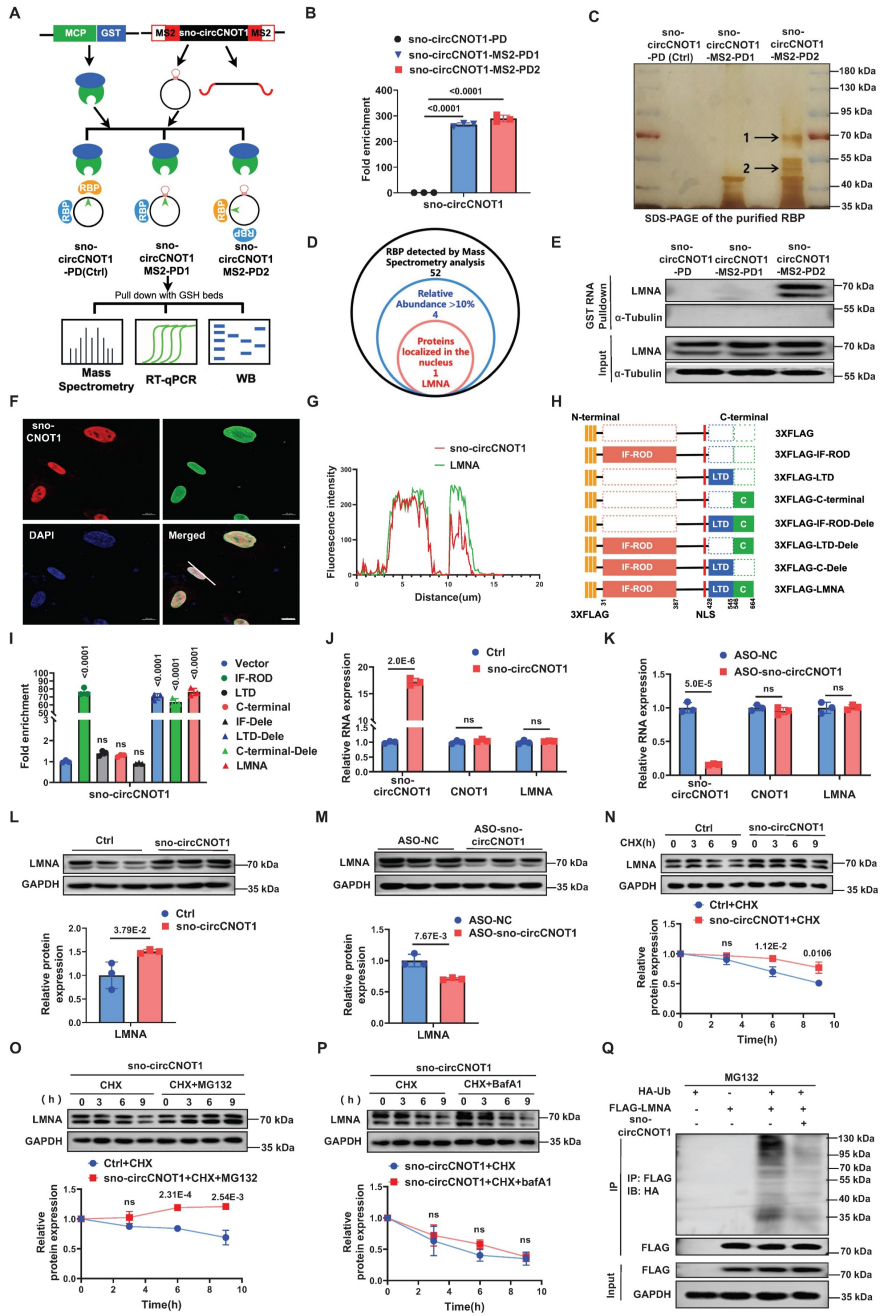
** sno-circCNOT1 interacts with LMNA and promotes its protein stabilization. A,** Schematic of the MS2 and MS2-coat protein (MS2-MCP)-based circRNA pull-down assay. HUVECs were transduced with lentivirus encoding MCP-GST and either MS2-tagged sno-circCNOT1 transcripts (sno-circCNOT1-MS2-PD1 and sno-circCNOT1-MS2-PD2) or control sno-circCNOT1 lacking MS2 (sno-circCNOT1-PD, Ctrl). Note: MS2 elements are split to prevent linear transcript pulldown. sno-circCNOT1-MS2-PD1 harbors MS2 elements at the circRNA splice junction, while sno-circCNOT1-MS2-PD2 contains MS2 elements distal to the splice site. RNA-protein complexes were isolated using glutathione (GSH) magnetic beads for mass spectrometry, RT-qPCR, or immunoblotting. **B,** RT-qPCR confirmed enrichment of sno-circCNOT1 in pull-down fractions (n = 3). **C,** Silver-stained SDS-PAGE of proteins pulled down by circRNA variants in HUVECs. Unique bands enriched in sno-circCNOT1-MS2-PD2 group are marked (black arrows). **D,** A Venn diagram identifying lamin A/C (LMNA) as a nuclear sno-circCNOT1-interacting protein among LC-MS/MS-detected candidates with >10% relative abundance. **E,** Immunoblot validation of sno-circCNOT1-LMNA interaction in pull-down samples. **F and G,** Nuclear colocalization of sno-circCNOT1 (Cy3-labeled probe, red) and LMNA (green) by fluorescence in situ hybridization (FISH) and immunofluorescence (IF). Nuclei: DAPI (blue). Scale bar: 10μm. **H,** Domain architecture of 3XFLAG-tagged LMNA and truncated mutants. **I,** RT-qPCR analysis of endogenous sno-circCNOT1 co-precipitated with FLAG-tagged LMNA truncations in HUVECs. n = 3. **J and K,** Overexpression (**J**) or knockdown (**K**) of sno-circCNOT1 does not alter mRNA levels of LMNA or CNOT1 (RT-qPCR). n = 3. **L and M,** immunoblot shows LMNA protein upregulation upon sno-circCNOT1 overexpressing (**L**) and downregulation after sno-circCNOT1 knockdown (**M**). Quantification normalized to GAPDH (n = 3). **N through P,** LMNA degradation assays in sno-circCNOT1-overexpressing HUVECs treated with: cycloheximide (CHX, 20 μM; **N**), CHX + proteasome inhibitor MG132 (20 μM; **O**), or CHX + lysosome inhibitor BafA1 (0.1 μM; **P**). n = 3. **Q,** Reduced ubiquitination of LMNA in sno-circCNOT1-overexpressing cells (IP). n = 3. Statistical analyses were performed using an unpaired two-tailed Student's t-test (**J** to **P**) or one-way ANOVA with Tukey's post hoc test (**B** and **I**). Data are presented as mean ± SEM. *P* values indicated. **A** and **H** were created by using Figdraw.

circRNAs exert their biological effects through multiple mechanisms, such as serving as miRNA sponges, binding to proteins, or being translated into short peptides 7,13. Given the exclusive nuclear localization of sno-circCNOT1, we hypothesized that it may exert its function through interactions with nuclear proteins. To identify potential binding partners, we performed MS2 RNA pull-down assays using three constructs: sno-circCNOT1-MS2-PD1 (MS2 hairpins inserted at the splice site, disrupting the back-spliced junction formation), sno-circCNOT1-MS2-PD2 (MS2 hairpins inserted distally, preserving the back-spliced junction), and a control sno-circCNOT1-PD (lacking MS2 hairpins) (Figure 3A). To minimize nonspecific pull-down of linear transcripts, the MS2 sequences were split and inserted upstream and downstream of sno-circCNOT1 (Figure 3A), ensuring that only back-spliced RNA would form an intact MS2 hairpin, thus enabling specific enrichment. By co-expressing MCP-GST (MS2 coat protein fused to glutathione-S-transferase) and using glutathione (GSH) magnetic beads, we selectively pulled down sno-circCNOT1 and its associated proteins. RT-qPCR confirmed specific enrichment of MS2-tagged sno-circCNOT1 (Figure 3B). Notably, compared to the other two groups, the sno-circCNOT1-MS2-PD2 group exhibited two distinct and significantly stronger protein bands at approximately 50 kDa and 70 kDa, as indicated by black arrows in Figure 3C. Mass spectrometry analysis of these bands identified 52 potential binding candidates (Figure 3D). Among these, four proteins showed relative abundances exceeding 10%, but only LMNA (laminin A/C) displayed nuclear localization, making it an attractive candidate for further investigation (Figure 3D; Supplementary Excel 1).

Western blot analysis of pull-down proteins confirmed specific detection of LMNA in the sno-circCNOT1-MS2-PD2 group, indicating that LMNA selectively binds sno-circCNOT1 when its back-spliced junction is intact (Figure 3E). Combined FISH and immunofluorescence staining further confirmed the nuclear co-localization of sno-circCNOT1 and LMNA (Figure 3F and 3G). To identify the interacting LMNA domain, we expressed FLAG-tagged full-length and truncated LMNA variants and assessed their binding to endogenous sno-circCNOT1 in ECs (Figure 3H). RNA immunoprecipitation (RIP) assays using anti-FLAG beads revealed that the IF-ROD domain of LMNA is both necessary and sufficient for interaction with sno-circCNOT1 (Figure 3I). Consistently, molecular docking identified a high-confidence interaction interface between the LMNA IF-ROD domain and the sno-circCNOT1 back-splice junction (Figure S7). Collectively, these findings demonstrate that LMNA interacts with sno-circCNOT1 through its IF-ROD domain, and that this interaction is dependent on the integrity of the back-spliced junction of sno-circCNOT1.

Next, we investigated whether sno-circCNOT1 influences LMNA expression. Overexpression or knockdown of sno-circCNOT1 had no effect on LMNA mRNA levels (Figure 3J and 3K). However, sno-circCNOT1 overexpression increased LMNA protein expression, whereas its knockdown decreased LMNA protein expression (Figure 3L and 3M). To determine whether sno-circCNOT1 enhances LMNA protein stability, we treated sno-circCNOT1-transduced ECs with cycloheximide (CHX) to inhibit protein synthesis and monitored the decay of LMNA protein over time. We found that sno-circCNOT1 overexpression prolonged LMNA protein stability (Figure 3N). Furthermore, treatment with the proteasome inhibitor MG132—but not with the lysosomal inhibitor bafilomycin A1 (BafA1)—effectively blocked LMNA degradation, suggesting that sno-circCNOT1 regulates LMNA stability via a proteasome-dependent pathway (Figure 3O and 3P). To investigate whether sno-circCNOT1 inhibits LMNA ubiquitination, HA-tagged ubiquitin (HA-Ub) and FLAG-tagged LMNA were co-transduced into ECs, followed by MG132 treatment. Immunoprecipitation assays revealed that sno-circCNOT1 overexpression significantly reduced LMNA ubiquitination (Figure 3Q), demonstrating that sno-circCNOT1 stabilizes LMNA by blocking its ubiquitination.

### sno-circCNOT1 promotes endothelial pyroptosis and inflammation via LMNA

**Figure 4:**
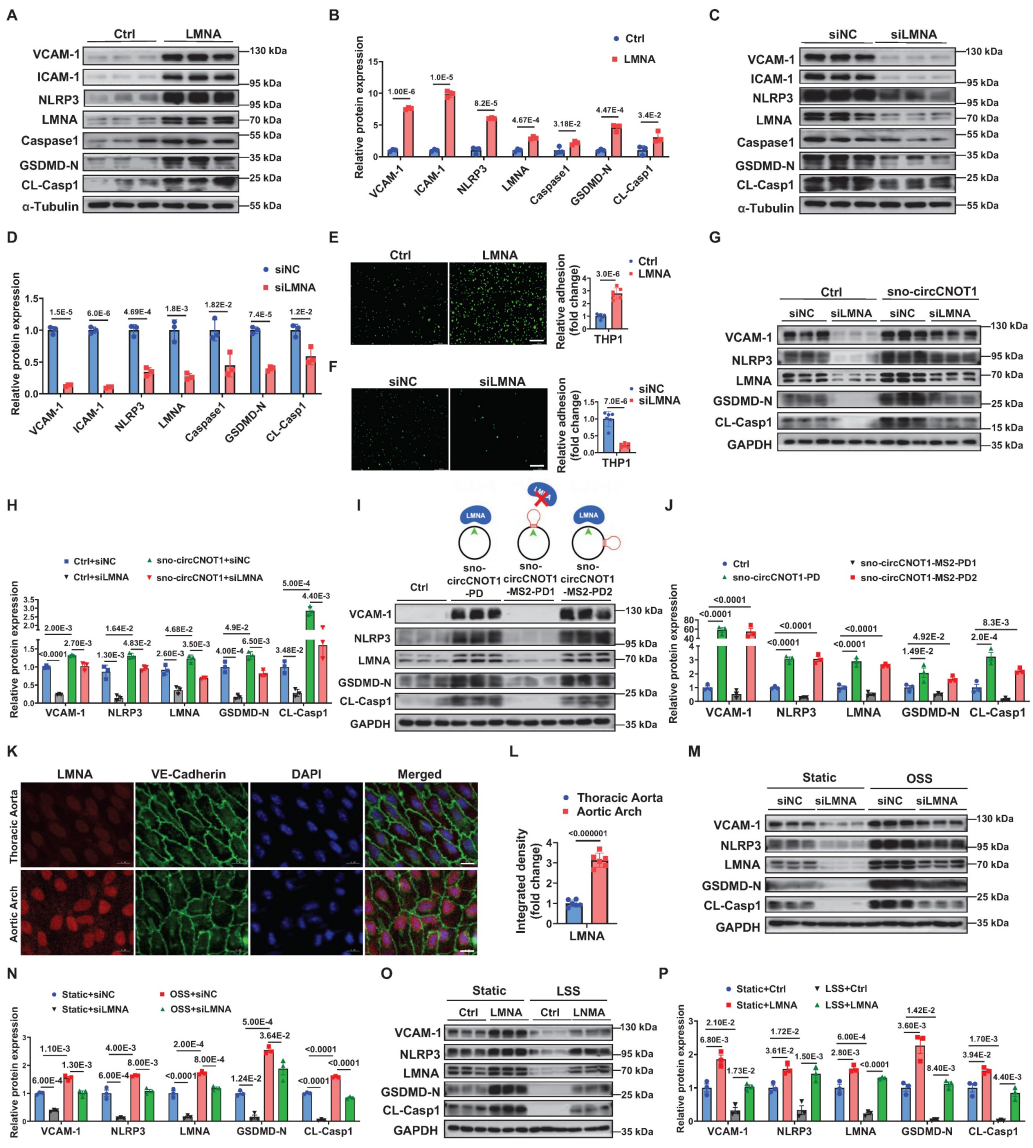
** Sno-circCNOT1 promotes endothelial pyroptosis and inflammation via LMNA. A through D,** Western blot analysis of inflammatory markers (VCAM-1 and ICAM-1) and pyroptosis-associated proteins (NLRP3, Caspase1, GSDMD-N, and cleaved CL-Casp1) in LMNA-overexpressing (**A and B**) or LMNA-knockdown (**C and D**) HUVECs. **E and F,** Monocyte adhesion assays using fluorescent THP-1 cells incubated with LMNA-overexpressing (**E**) or LMNA-knockdown (**F**) HUVECs. Scale bar = 200 μm. *n* = 6. **G and H,** HUVECs transduced with PLO5-ciR (Ctrl) or sno-circCNOT1 lentivirus for 24 hours followed by transfection with LMNA siRNA (siLMNA) or control siRNA (siNC) for 48 hours. Protein levels of LMNA, VCAM-1, NLRP3, GSDMD-N, and CL-Casp1 were determined *via* western blot. **I and J,** (**I**) Schematic of wild-type sno-circCNOT1 (sno-circCNOT1-PD), MS2 splice-site mutant disrupting LMNA binding (sno-circCNOT1-MS2-PD1), and distal MS2 mutant retaining LMNA binding (sno-circCNOT1-MS2-PD2). The green line indicates the splicing site. Western blot analysis of VCAM-1, NLRP3, LMNA, GSDMD-N, and CL-Casp1 in HUVECs transduced with control (Ctrl), sno-circCNOT1-PD, sno-circCNOT1-MS2-PD1, or sno-circCNOT1-MS2-PD2 lentivirus. (**J**). Quantification of proteins normalized to GAPDH. n = 3. **K and L,** En face immunofluorescence showing elevated endothelial LMNA expression in mouse aortic arch (exposed to atheroprone shear stress) versus thoracic aorta (exposed to atheroprotective shear stress). Scale bar = 10 μm. (n = 6). **M and N,** HUVECs transfected with LMNA siRNA (siLMNA), or control siRNA (siNC) for 48 hours were exposed to atheroprone OSS (0.5 ± 4 dyne/cm^2^ at 1 Hz) for 24 hours. Protein levels of LMNA, VCAM-1, NLRP3, GSDMD-N and CL-Casp1 were analyzed by western blot. **O and P,** HUVECs transduced with LMNA or mCherry (control, Ctrl) lentivirus for 48 hours were subjected to atheroprotective LSS (15 dyne/cm^2^) for 24 hours. Western blot analysis of LMNA, VCAM-1, NLRP3, GSDMD-N and CL-Casp1 is shown. Statistical analyses were conducted using an unpaired two-tailed Student's t-test (**A** to** F** and **K** to **L**) or one-way ANOVA (**I** to** J**) or two-way ANOVA (**G** to **H** and **M** to **P**) with Tukey's post hoc test. Data are presented as mean ± SEM. *P* values indicated.

Next, we investigated whether LMNA mediates sno-circCNOT1-induced endothelial pyroptosis and inflammation. As expected, LMNA overexpression in ECs significantly increased expression of pyroptotic and inflammatory markers (VCAM-1, ICAM-1, NLRP3, Caspase1, GSDMD-N and CL-Casp1), while LMNA silencing reduced their expression (Figure 4A through 4D). Consistent with these molecular changes, LMNA overexpression enhanced monocyte adhesion to ECs, whereas LMNA silencing attenuated this adhesion (Figure 4E and 4F). Notably, LMNA knockdown significantly reversed the pro-pyroptotic and pro-inflammatory effects triggered by sno-circCNOT1 overexpression (Figure 4G and 4H), suggesting that sno-circCNOT1 acts through LMNA. Furthermore, disruption of LMNA binding by inserting an MS2 RNA hairpin at the back-spliced junction of sno-circCNOT1 completely abolished its pro-pyroptotic and pro-inflammatory phenotypes (Figure 4I and 4J). These results further support the conclusion that sno-circCNOT1 promotes endothelial pyroptosis and inflammation *via* LMNA-mediated mechanisms.

We next investigated whether shear stress modulates LMNA expression. En face immunofluorescence staining revealed higher LMNA expression in the lesser curvature of the aortic arch-an area exposed to atheroprone shear stress-compared to the thoracic aorta, which is subjected to atheroprotective shear stress (Figure 4K and 4L). Rescue experiments showed that LMNA silencing significantly attenuated OSS-induced upregulation of pyroptotic and inflammatory markers in ECs (Figure 4M and 4N). Similarly, LMNA overexpression counteracted the anti-pyroptotic and anti-inflammatory effects of LSS (Figure 4O and 4P). Collectively, these findings indicate that shear stress regulates endothelial pyroptosis and inflammation through the sno-circCNOT1/LMNA axis.

### LMNA exacerbates endothelial pyroptosis and inflammation by stabilizing METTL14

**Figure 5:**
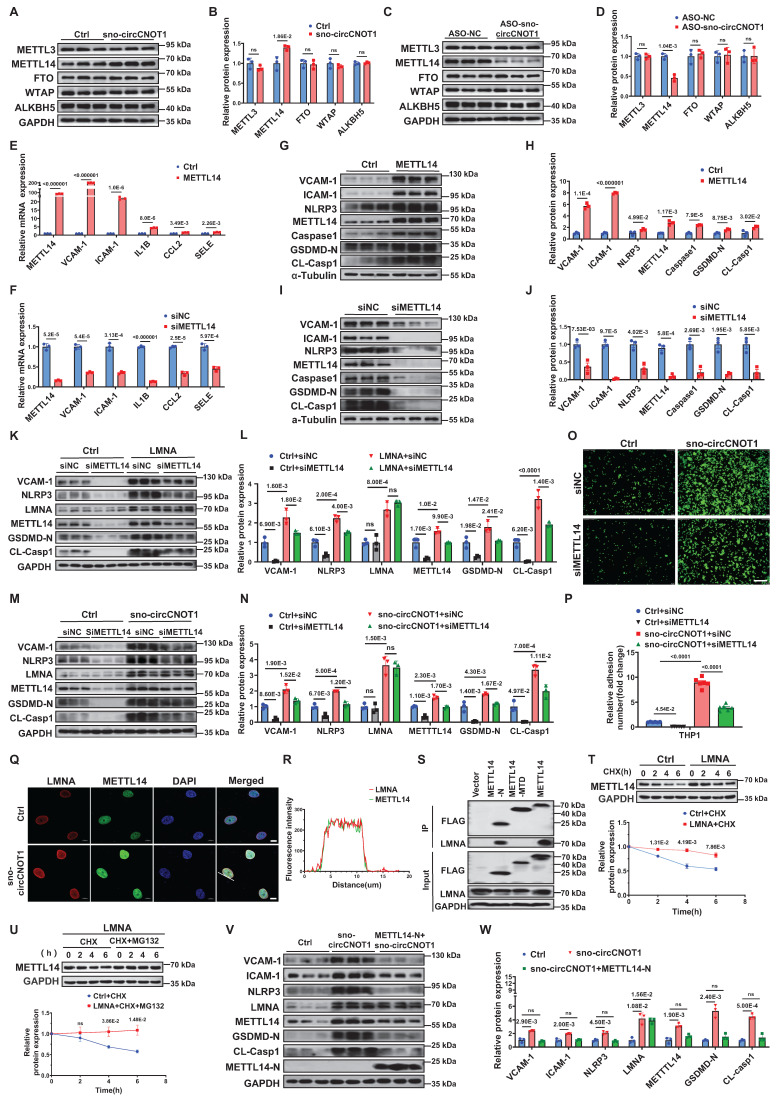
** LMNA exacerbates endothelial pyroptosis and inflammation by stabilizing METTL14 protein expression. A through D,** Western blot analysis of METTL3, METTL14, FTO, WTAP and ALKBH5 in sno-circCNOT1-overexpressing (**A and B**) or knockdown (**C and D**) HUVECs. n = 3. **E and F,** RT-qPCR quantification of METTL14, VCAM-1, ICAM-1, IL1B, CCL2 and SELE mRNA levels in METTL14-overexpressing (**E**) or knockdown (**F**) HUVECs, normalized to GAPDH. n = 3. **G through J,** Western blot analysis of METTL14, VCAM-1, ICAM-1, NLRP3, Caspase1, GSDMD-N, and CL-Casp1 in HUVECs with METTL14 overexpression (**G and H**) or METTL14 knockdown (**I and J**). n = 3. **K and L,** HUVECs transduced with LMNA or control (Ctrl) lentivirus for 24 hours were subsequently transfected with METTL14 siRNA (siMETTL14) or control siRNA (siNC) for another 48 hours. Protein levels of VCAM-1, NLRP3, LMNA, METTL14, GSDMD-N, and CL-Casp1 were determined using western blot. n = 3. **M and N,** HUVECs transduced with Ctrl or sno-circCNOT1 lentivirus for 24 hours were subsequently transfected with siMETTL14 or siNC for 48 hours. Protein levels of VCAM-1, NLRP3, LMNA, METTL14, GSDMD-N, and CL-Casp1 were determined using western blot. **O and P,** Monocyte adhesion assays using fluorescently labeled THP-1 cells co-cultured with HUVECs that were first transduced with Ctrl or sno-circCNOT1 lentivirus for 24 h, followed by transfection with siMETTL14 or siNC for an additional 48 h. Scale bar = 200 μm. n = 6. **Q and R,** Immunofluorescence (IF) imaging showing co-localization and increased expression of LMNA (red) and METTL14 (green) in the nucleus of sno-circCNOT1-transduced HUVECs. Scale bar = 10 μm. **S,** Western blot analysis of LMNA in anti-FLAG immunoprecipitates from HUVECs expressing FLAG-tagged METTL14 truncations. Input lysates are shown. **T and U,** Western blot analysis of METTL14 protein stability in LMNA-overexpressing HUVECs treated with cycloheximide (CHX; 20μM) (**T**) or CHX + MG132 (20μM) (**U**) for the indicated durations. **V and W,** Western blot analysis of indicated proteins in HUVECs transduced with sno-circCNOT1 or METTL14-N plus sno-circCNOT1 lentivirus for 72 hours. Statistical analyses were conducted using the unpaired two-tailed Student's t-test (**A** to **J** and **T** to **U**) or two-way analysis of variance (ANOVA) with Tukey's post hoc test (**K** to **P** and **V** to **W**). Data are presented as mean ± SEM. *P* values indicated.

N6-methyladenosine (m⁶A), the predominant internal modification of mRNA in eukaryotes, plays crucial roles in various biological processes, including pyroptosis and inflammation 14-17. Previous studies implicate m⁶A modification in shear stress-mediated regulation of endothelial inflammation 18,19. Given that LMNA is predominantly localized in the nucleus—the primary site of m⁶A modification—we investigated whether LMNA exerts its biological effects through the modulation of m⁶A. Intriguingly, manipulation of sno-circCNOT1 expression did not significantly alter the mRNA or protein levels of key m⁶A regulatory components, including the methyltransferase methyltransferase-like 3 (METTL3), the auxiliary protein Wilms tumor 1-associated protein (WTAP), and the demethylases fat mass and obesity-associated protein (FTO) and AlkB homolog 5 (ALKBH5) (Figure 5A-5D; Figure S8). However, it selectively modulated METTL14 protein expression without altering its mRNA levels (Figure 5A-5D; Figure S3). Notably, METTL14 functions as a critical adaptor that facilitates RNA substrate recognition by METTL3, and the METTL3-METTL14 heterodimer is essential for enzymatic m⁶A deposition 20,21. To determine whether METTL14 mediates the pro-pyroptotic and pro-inflammatory effects of LMNA, we conducted functional analyses. Overexpression of METTL14 induced pro-pyroptotic and pro-inflammatory phenotypes (Figure 5E, 5G and 5H), whereas its silencing had the opposite effects (Figure 5F, 5I and 5J). These results were further supported by monocyte adhesion assays (Figure S9). Importantly, METTL14 silencing significantly attenuated the pro-pyroptotic and pro-inflammatory effects triggered by LMNA (Figure 5K and 5L), sno-circCNOT1 (Figure 5M and 5N), or OSS (Figure S10). Furthermore, METTL14 suppression mitigated sno-circCNOT1-induced monocyte adhesion to ECs (Figure 5O and 5P). Collectively, these findings suggest that OSS-induced sno-circCNOT1 modulates endothelial pyroptosis and inflammation through the LMNA/METTL14 axis.

Next, we investigated the mechanism by which LMNA regulates METTL14 protein expression. We first observed that sno-circCNOT1 overexpression promoted the protein expression of both LMNA and METTL14, as well as their nuclear colocalization (Figure 5Q and 5R), suggesting a potential interaction between the two proteins. To further characterize this interaction, we examined the binding capacity of FLAG-tagged full-length and truncated METTL14 proteins to endogenous LMNA. Western blot analysis revealed that the N-terminal domain of METTL14 (METTL14-N) is both necessary and sufficient for interaction with LMNA (Figure 5S). Given this interaction, we next explored whether LMNA modulates METTL14 protein stability. As anticipated, LMNA overexpression significantly enhanced METTL14 stability (Figure 5T). Moreover, treatment with the proteasome inhibitor MG132 (Figure 5U), but not the lysosomal inhibitor BafA1(Figure S11), effectively prevented METTL14 degradation, indicating that LMNA promotes METTL14 stabilization *via* a proteasome-dependent pathway. Since sno-circCNOT1 upregulates LMNA expression, we further examined its impact on METTL14 stability. Consistently, sno-circCNOT1 overexpression promotes METTL14 stability through the proteasome-dependent mechanism (Figure S12). Importantly, overexpression of the METTL14-N domain, which disrupts LMNA signaling, markedly reduced endogenous METTL14 expression and the pro-pyroptotic/pro-inflammatory phenotypes induced by either sno-circCNOT1 overexpression or OSS, while LMNA expression remained unchanged (Figure 5V and 5W; Figure S13 and S14). These findings collectively demonstrate that the LMNA-METTL14 interaction stabilizes METTL14 protein, thereby promoting endothelial pyroptosis and inflammation.

### METTL14-dependent m⁶A modification of NLRP3 mRNA mediates the pro-pyroptotic and pro-inflammatory effects of sno-circCNOT1

**Figure 6:**
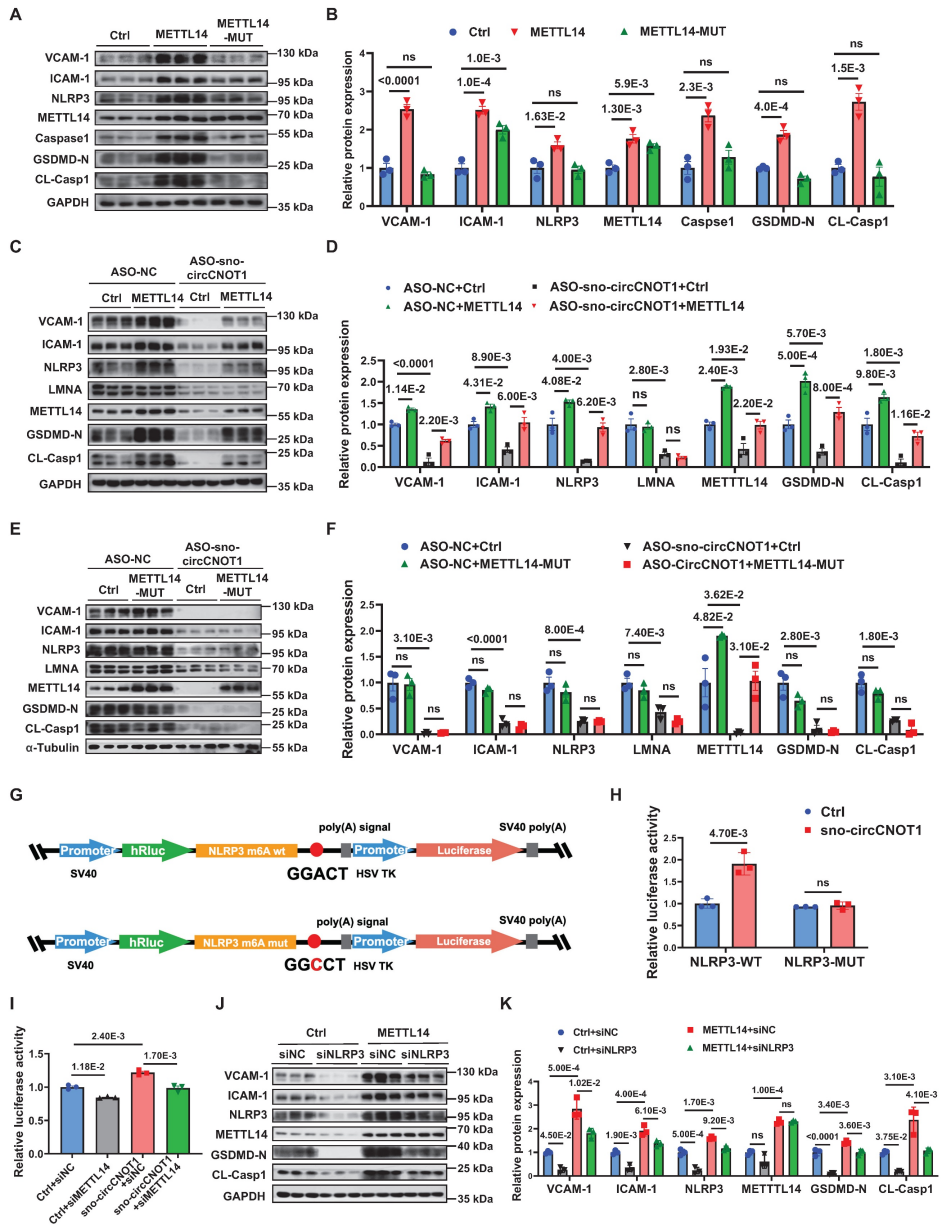
** METTL14-dependent m6A modification of NLRP3 mRNA mediates the pro-pyroptotic and pro-inflammatory effects of sno-circCNOT1. A and B,** Western blot analysis of VCAM-1, ICAM-1, NLRP3, METTL14, Caspase-1, GSDMD-N and CL-Casp1 in HUVECs transduced with control (Ctrl), wild-type METTL14 (METTL14-WT), or functionally impaired METTL14 R298P mutant (METTL14-MUT) lentivirus for 72 hours. n = 3. **C through F,** HUVECs transduced with Ctrl, METTL14-WT (**C and D**), or METTL14-MUT (**E and F**) lentivirus for 24 hours, followed by transfection with ASO-NC (control) or ASO-sno-circCNOT1 for 48 hours. Western blot analysis of VCAM-1, ICAM-1, NLRP3, LMNA, METTL14, GSDMD-N and CL-Casp1. n = 3. **G,** Schematic of wild-type (NLRP3-WT) and m6A site-mutated (NLRP3-MUT) luciferase reporter constructs. **H,** Luciferase activity of NLRP3-WT or NLRP3-MUT in Lenti-X 293T cells co-transfected with sno-circCNOT1 or Ctrl plasmids was measured using the Dual-Luciferase Reporter Assay System. n = 3. **I,** Relative luciferase activity of NLRP3-WT in LentiX-293 T cells transfected with sno-circCNOT1 or Ctrl plasmids, followed by transfection with METTL14-targeting siRNA (siMETTL14) or control siRNA (siNC). Renilla luciferase activity was normalized to firefly luciferase activity. **J and K,** Western blot analysis of VCAM-1, ICAM-1, NLRP3, METTL14, GSDMD-N and CL-Casp1 in HUVECs transduced with METTL14-overexpressing or control lentivirus for 24 hours, followed by transfection with NLRP3-targeting siRNA (siNLRP3) or siNC for 48 hours. n = 3. Statistical analyses were performed using the unpaired two-tailed Student's t-test (**H**) or one-way ANOVA (**A** to **B**) or two-way ANOVA (**C** to **F** and **I** to **K**) with Tukey's post hoc test. *P* values indicated. **G** was created by using Figdraw.

Given that the R298P mutation in METTL14 markedly reduces the methyltransferase activity of the METTL3-METTL14 heterodimer 21, we generated a vector expressing the METTL14 R298P mutant (METTL14-MUT) to evaluate its functional dependence on m⁶A modification. As anticipated, overexpression of wild-type METTL14, but not METTL14-MUT, significantly promoted pro-pyroptotic and pro-inflammatory phenotypes in ECs (Figure 6A and 6B). Furthermore, wild-type METTL14 overexpression (Figure 6C and 6D), but not METTL14-MUT (Figure 6E and 6F), abrogated the anti-pyroptotic and anti-inflammatory effects induced by sno-circCNOT1 knockdown. These results suggest that sno-circCNOT1 promotes endothelial pyroptosis and inflammation through an m⁶A-dependent mechanism.

Next, we aimed to identify the downstream effectors mediating the biological functions of METTL14. NLRP3, a key regulator of endothelial pyroptosis and inflammation, was confirmed as a direct downstream target of METTL14 22. Supporting this, wild-type METTL14 overexpression markedly elevated NLRP3 protein levels (Figure 6A), whereas METTL14-MUT had no such effect. Furthermore, METTL14 overexpression (Figure 6C and 6D), but not METTL14-MUT (Figure 6E and 6F), effectively restored NLRP3 protein levels suppressed by sno-circCNOT1 knockdown, supporting the notion that METTL14 regulates NLRP3 expression through an m⁶A-dependent mechanism. Focusing on a representative m⁶A site in NLRP3 mRNA 22, dual-luciferase reporter assays demonstrated that overexpression of sno-circCNOT1 significantly enhanced the luciferase activity of wild-type *NLRP3* reporter (NLRP3-WT), but not that of the m⁶A motif mutant (NLRP3-MUT) (Figures 6G and 6H). Importantly, METTL14 knockdown abolished the sno-circCNOT1-induced increase in NLRP3-WT luciferase activity (Figure 6I), indicating that sno-circCNOT1 regulates NLRP3 expression via METTL14-mediated m⁶A modification of NLRP3 mRNA. To further validate this pathway, we performed a rescue experiment by silencing NLRP3 and observed a partially reversal of the pro-pyroptotic and pro-inflammatory phenotypes induced by METTL14 overexpression (Figure 6J and 6K). Collectively, these findings suggest that sno-circCNOT1 modulates endothelial pyroptosis and inflammation through the LMNA/METTL14/NLRP3 axis in an m⁶A-dependent manner.

### Endothelial sno-circCNOT1 aggravates atherosclerosis in *ApoE⁻/⁻* mice

**Figure 7:**
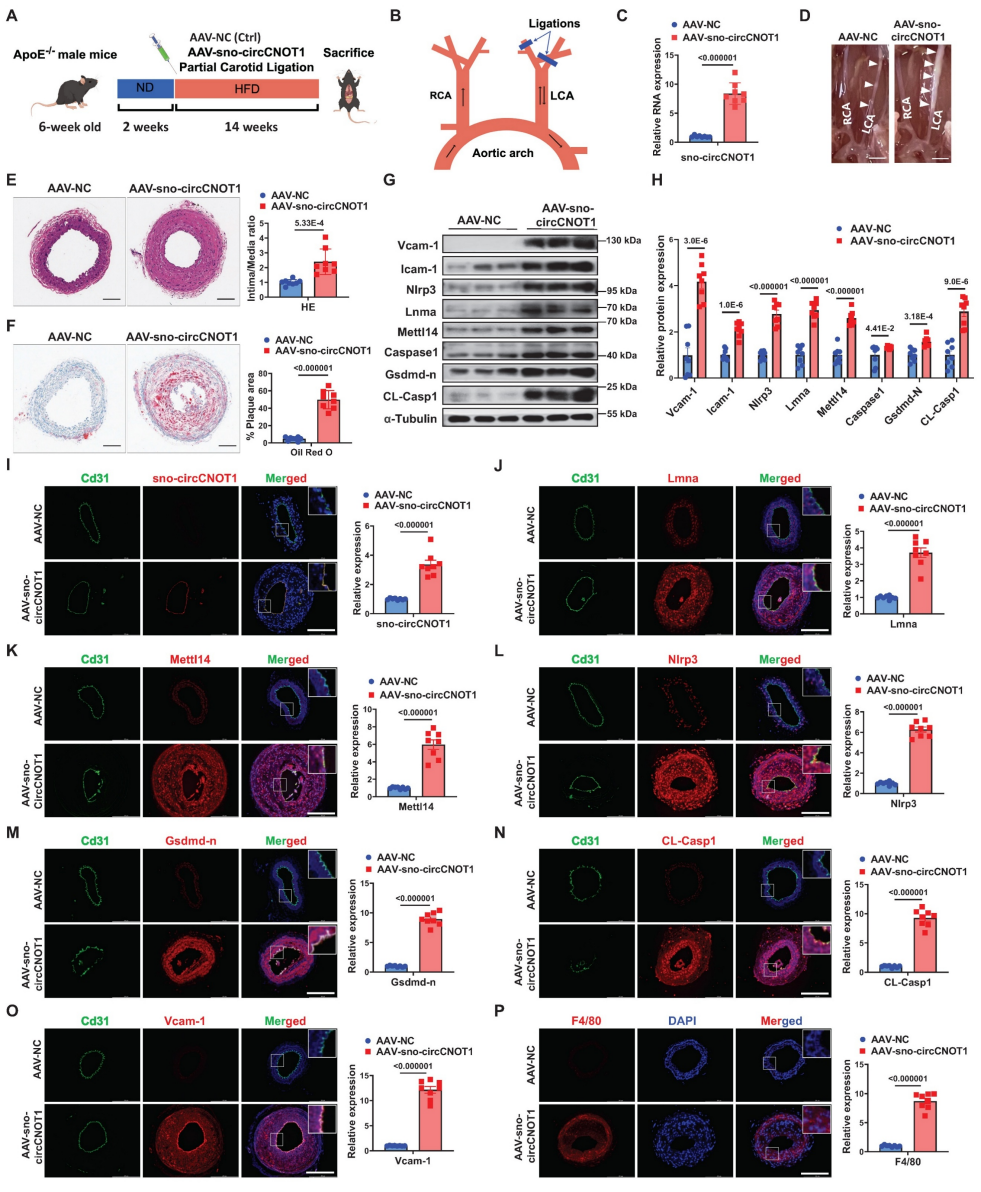
** Endothelial sno-circCNOT1 aggravates atherosclerosis in *ApoE⁻/⁻* mice. A,** Schematic of the *in vivo* experimental workflow. **B,** Schematic illustration of partial carotid artery ligation. RCA, right common carotid artery. LCA, left common carotid artery. **C,** RT-qPCR analysis of sno-circCNOT1 expression in mouse aortic tissues. n = 8. **D,** Representative images of LCA atherosclerotic plaques (white arrows). **E,** Representative hematoxylin and eosin (H&E)-stained LCA sections (left) and plaque area quantification (right). Scale bar: 100 μm. n = 8. **F,** Representative Oil Red O-stained LCA sections (left) and lipid deposition quantification (right). Scale bar: 100 μm. n = 8. **G and H,** Western blotting analysis of Vcam-1, Icam-1, Nlrp3, Lmna, Mettl14, Caspase-1, Gsdmd-n CL-Casp1, and FLAG-Mettl14-N in aortic tissues from mice injected with AAV-sno-circCNOT1 or AAV-NC. **I,** Representative FISH images showing endothelial overexpression of sno-circCNOT1 (red) in LCA endothelium. CD31 (green) marks endothelial cells. Scale bar: 200 μm. **J through P,** Representative immunofluorescence staining of Lmna (**J**), Mettl14 (**K**), Nlrp3 (**L**), Gsdmd-n (**M**), CL-Casp1 (**N**), Vcam-1 (**O**), F4/80 (**P**), and Cd31 (endothelial marker) in the ligated LCA sections. Nuclei were counterstained with DAPI (blue). Scale bar: 200 μm. Quantification data are shown on the right. n = 8. Scale bar: 200 μm. Statistical analyses were conducted using the unpaired two-tailed Student's t-test. Data are presented as mean SEM,* n* = 8 mice per group. *P* values indicated. **A** and **B** were created by using Figdraw.

To investigate the role of sno-circCNOT1 *in vivo*, *ApoE⁻/⁻* mice were injected in situ with either a sno-circCNOT1 adeno-associated virus (AAV-sno-circCNOT1) or a control virus (AAV-NC). The mouse Icam2 promoter was utilized to drive endothelial-specific expression of sno-circCNOT1. Subsequently, the mice underwent partial ligation of the left common carotid artery (LCA) and were fed a high-fat diet (HFD) (Figure 7A and 7B). There were no significant differences in serum triglyceride (TG), cholesterol (TC), and low-density lipoprotein cholesterol (LDL-C) levels between AAV-sno-circCNOT1 and AAV-NC groups (Figure S15). RT-qPCR confirmed successful overexpression of sno-circCNOT1 (Figure 7C). Endothelial sno-circCNOT1 overexpression led to increased atherosclerotic plaque formation compared to control groups (Figure 7D; Figure S16). ApoB immunostaining confirmed that endothelial overexpression of sno-circCNOT1 increased lipid deposition within carotid lesions (Figure S17). These findings were further supported by histological analyses, including hematoxylin and eosin (H&E) staining (Figure 7E) and Oil Red O staining (Figure 7F). Additionally, western blot analysis demonstrated that sno-circCNOT1 overexpression upregulated the protein levels of Lmna, Mettl14 and Nlrp3, along with elevated expression of pyroptosis and inflammation-related markers in mouse aortic tissues (Figure 7G and 7H). Furthermore, enzyme-linked immunosorbent assay (ELISA) demonstrated elevated levels of pro-inflammatory cytokines, including IL-1β, IL-18, and monocyte chemoattractant protein-1 (Mcp1), in the sno-circCNOT1 group compared to controls (Figure S18). FISH assays confirmed the endothelial-specific sno-circCNOT1 overexpression in the mouse LCA (Figure 7I), consistent with the RT-qPCR results. Immunofluorescence staining further showed that sno-circCNOT1 upregulated the protein levels of Lmna (Figure 7J), Mettl14 (Figure 7K), Nlrp3 (Figure 7L), Gsdmd-N (Figure 7M), CL-Casp1 (Figure 7N), Vcam-1 (Figure 7O), as well as the macrophage marker F4/80 (Figure 7P) and CD68 (Figure S19). Taken together, these findings indicate that endothelial sno-circCNOT1 overexpression exacerbates atherosclerosis by promoting vascular pyroptosis and inflammation.

### Endothelial METTL14-N overexpression attenuates atherosclerosis in *ApoE⁻/⁻* mice

**Figure 8:**
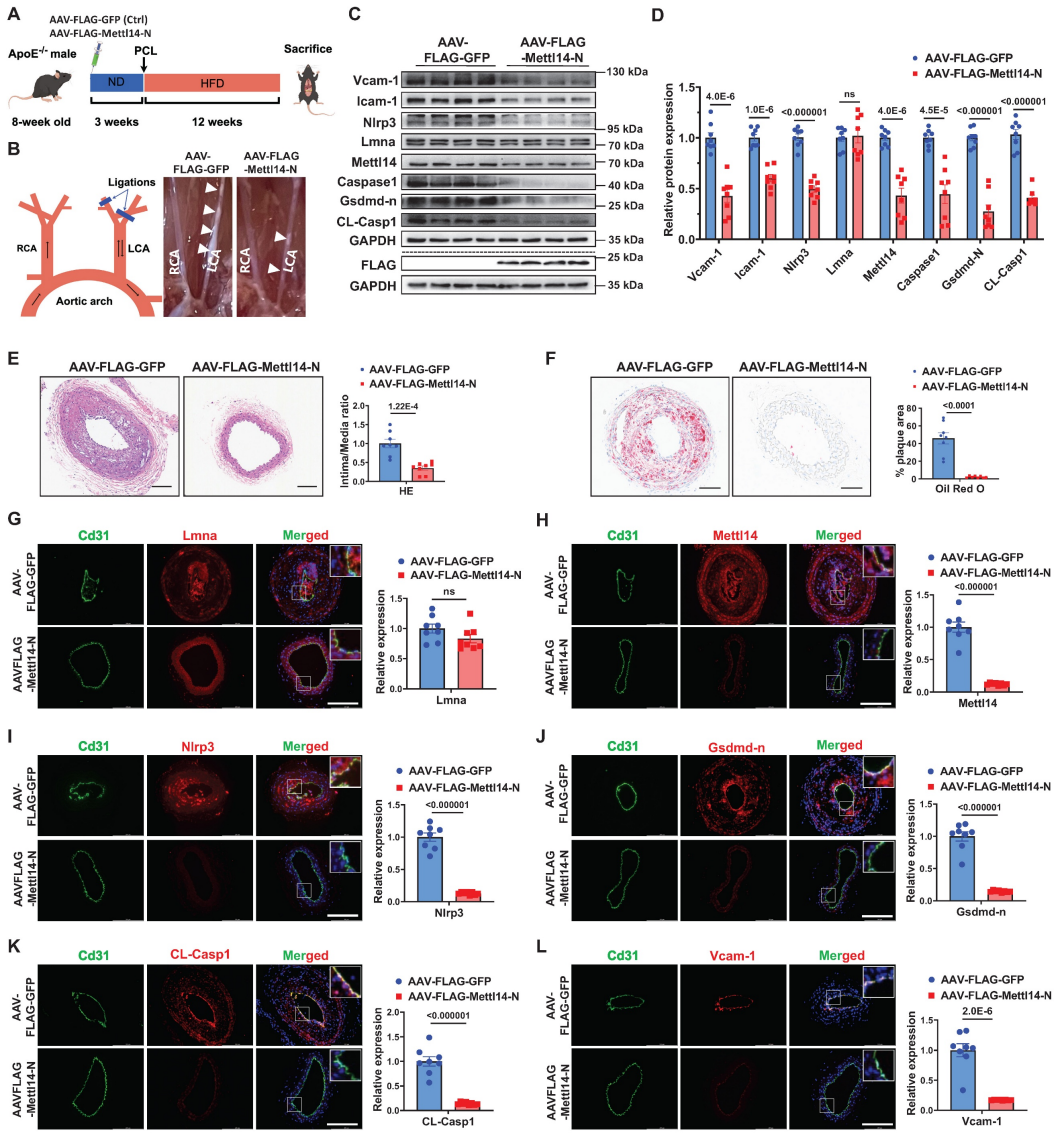
** Endothelial METTL14-N overexpression attenuates atherosclerosis in *ApoE⁻/⁻* mice. A,** Schematic of the *in vivo* experimental workflow. **B,** Schematic illustration of partial carotid artery ligation and representative images of LCA atherosclerotic plaques (white arrows). PCL, partial ligation of the left carotid artery. RCA, right common carotid artery. LCA, left common carotid artery.** C and D,** Western blot analysis of Vcam-1, Icam-1, Nlrp3, Lmna, Mettl14, Caspase-1, Gsdmd-n and CL-Casp1 in aortic tissues from mice injected with AAV-FLAG-Mettl14-N or AAV-FLAG-GFP. n = 8. **E,** Representative hematoxylin and eosin (H&E)-stained LCA sections (left) and plaque area quantification (right). Scale bar: 100 μm. n = 8. **F,** Representative Oil Red O-stained LCA sections (left) and lipid deposition quantification (right). Scale bar: 100 μm. n = 8. **G through L,** Representative immunofluorescence staining of Lmna (**G**), Mettl14 (**H**), Nlrp3 (**I**), Gsdmd-n (**J**), CL-Casp1 (**K**), and Vcam-1 (**L**) in the ligated LCA sections. Cd31 (green) marks endothelium; Nuclei counterstained with DAPI (blue). Scale bar: 200 μm. Quantification data are shown on the right. n = 8. Statistical significance was determined by unpaired two-tailed Student's t-test (**C** to **L**). Data are presented as mean SEM,* n* = 8 mice per group. *P* values indicated.

To evaluate the therapeutic potential of METTL14-N *in vivo*, *ApoE⁻/⁻* mice were intravenously administered AAV vectors encoding either FLAG-tagged mouse Mettl14-N (AAV-FLAG-Mettl14-N) or a FLAG-tagged GFP control (AAV-FLAG-GFP). Endothelial-specific expression was driven by the *Icam2* promoter. Atherosclerosis was induced by partial ligation of the LCA, followed by a 12-week HFD (Figure 8A). Immunofluorescence staining confirmed that AAV-FLAG-Mettl14-N achieved efficient and specific endothelial expression *in vivo* (Figure S20). There were no significant differences in serum triglyceride (TG), cholesterol (TC), or low-density lipoprotein cholesterol (LDL-C) levels between the Mettl14-N overexpression and control groups (Figure S21). Western blot analysis confirmed successful overexpression of Mettl14-N (AAV-FLAG-Mettl14-N), which was associated with reduced protein levels of endogenous Mettl14, as well as decreased expression of pyroptosis-related markers (Nlrp3, Gsdmd-N, and CL-Casp1) and inflammatory markers (Vcam-1 and Icam-1) in aortic tissues from the Mettl14-N treatment group (Figure 8C and 8D). Compared to controls, Mettl14-N overexpression significantly suppressed atherosclerotic plaque formation (Figure 8B; Figure S22A). These findings were corroborated by histopathological assessments using H&E staining (Figure 8E) and lipid deposition quantification via Oil Red O staining (Figure 8F). Furthermore, ELISA analysis revealed lower serum levels of proinflammatory cytokines (IL-1β, IL-18, and Mcp1) in the METTL14-N overexpression group (Figure S22B through S22D). Immunofluorescence staining further confirmed that endothelial expression of Mettl14, Nlrp3, Gsdmd-N, CL-Casp1, Vcam-1, and F4/80 was downregulated in the METTL14-N overexpression group compared with the control group, while Lmna remained unaffected (Figure 8G-8L; Figure S23). Collectively, these results demonstrate that endothelial-specific METTL14-N overexpression attenuates atherosclerosis progression in *ApoE⁻/⁻* mice, highlighting its potential as a protein-based therapeutic strategy for treating atherosclerosis.

## Discussion

The relationship between shear stress and atherosclerosis has been extensively investigated 23-25; however, the precise molecular mechanisms by which shear stress regulates atherosclerosis through circRNAs remain incompletely understood. In this study, we identified a mechanosensitive snoRNA-like circRNA, sno-circCNOT1, which is downregulated under anti-atherosclerotic LSS but upregulated under pro-atherosclerotic OSS and IL-1β stimulation. This circRNA promotes atherosclerotic progression by enhancing endothelial pyroptosis and inflammation. Mechanistically, sno-circCNOT1 accumulates in the nucleus through its snoRNA-like motif and stabilizes the LMNA protein by binding to the LMNA IF-ROD domain, mediated by sequences spanning its back-splice junction. Furthermore, LMNA interacts with the N-terminal region of METTL14, thereby stabilizing METTL14 and promoting NLRP3 expression in an m⁶A-dependent manner. Critically, overexpression of METTL14-N consistently attenuates atherosclerosis progression *in vitro* and *in vivo* by disrupting the LMNA-METTL14 signaling pathway. These findings establish the sno-circCNOT1-LMNA-METTL14-NLRP3 axis as a promising therapeutic target and propose METTL14 N-terminal domain-based intervention as a novel anti-atherosclerotic strategy.

SnoRNAs are evolutionarily conserved non-coding RNAs with typical length of 60-300 nucleotides that primarily mediate post-transcriptional modifications of ribosomal RNAs 26. Recent studies have identified a novel subclass of long non-coding RNAs (lncRNAs), termed snoRNA-like lncRNAs, which incorporate snoRNA-derived sequences essential for their stability and subcellular localization 27-29. While the functions of snoRNA-like lncRNAs have been extensively characterized, the biological significance of snoRNA-like circRNAs remains largely unexplored. In this study, we identified sno-circCNOT1, a snoRNA-like circRNA containing SNORA50A-derived sequences. Notably, sno-circCNOT1 lacks the canonical 5′-guACAuuu-3′ tail sequence that includes the ACA motif, suggesting that it cannot be processed into a functional SNORA50A. Consistent with this structural feature, functional assays revealed that SNORA50A overexpression does not affect endothelial pyroptosis or inflammatory responses, thereby excluding the possibility that sno-circCNOT1 exerts its effects via SNORA50A. In line with previous findings on the role of snoRNA sequences in determining lncRNA subcellular distribution 28, we demonstrated that the snoRNA-like motif embedded within sno-circCNOT1 is essential for its nuclear retention. Moreover, deletion of this sequence completely abolished the pro-pyroptotic and pro-inflammatory activities of sno-circCNOT1, underscoring the indispensable role of SNORA50A-derived sequences in its function.

Lamin A and C, collectively referred to as lamin A/C, are A-type lamins generated from the *LMNA* gene through alternative splicing. These proteins are key components of the nuclear lamina, a structural scaffold essential for maintaining nuclear architecture and mechanical integrity. Beyond their structural functions, lamin A/C also regulates genome stability, chromatin organization, and gene expression 30-33. Pathogenic *LMNA* mutations or dysregulated lamin A/C expression can impair these multifunctional roles, leading to diverse diseases 34,35. In this study, we demonstrated that pro-atherosclerotic OSS upregulates sno-circCNOT1 and LMNA protein expression both *in vitro* and *in vivo*. sno-circCNOT1 stabilizes the LMNA protein by binding to the IF-ROD domain of LMNA via its unique back-spliced junction sequence. This interaction inhibits LMNA protein degradation through the ubiquitin-proteasome pathway. Notably, LMNA silencing significantly attenuated OSS-induced endothelial pyroptosis and inflammatory responses. Our findings reveal a novel regulatory axis in which sno-circCNOT1 mediated LMNA stabilization, providing mechanistic insights into how LMNA dysregulation contributes to atherosclerosis progression.

METTL14, a core component of the m⁶A methyltransferase complex, has been shown to promote endothelial pyroptosis and inflammation 36-40. Consistent with previous reports, our study demonstrates that METTL14 facilitates endothelial pyroptosis and inflammation by targeting NLRP3. Further analysis revealed that METTL14 enhances NLRP3 expression in an m⁶A-dependent manner. Notably, we identified a previously unrecognized interaction between LMNA and the N-terminal domain of METTL14, which appears to be critical for maintaining METTL14 stability. Intriguingly, overexpression of the METTL14 N-terminal fragment not only suppressed endogenous METTL14 levels but also effectively mitigated the pro-atherosclerotic effects mediated by sno-circCNOT1. Together, these findings uncover a novel function of the METTL14 N-terminal in the regulation of atherosclerosis and highlighting its potential as a protein-based therapeutic strategy for atherosclerosis intervention.

This study has several limitations. First, the precise mechanisms by which shear stress regulates sno-circCNOT1 expression warrant further investigation. Second, although we demonstrated that AAV-mediated, endothelial-specific METTL14-N overexpression attenuates atherosclerotic progression in *ApoE⁻/⁻* mice, the development of more advanced delivery systems, such as extracellular vesicle-based systems, for METTL14-N protein delivery is needed for future clinical applications. Third, although our *in vitro* and *in vivo* experiments demonstrate that sno-circCNOT1 promotes atherosclerosis progression through the LMNA/METTL14 /NLRP3 axis, its clinical significance remains to be fully elucidated. Subsequent investigations should validate endothelial-specific upregulation of sno-circCNOT1 in human atherosclerotic plaques.

In conclusion, our findings demonstrate that shear-sensitive sno-circCNOT1 promotes endothelial pyroptosis and inflammation *via* the LMNA/METTL14/NLRP3 axis, thereby accelerating atherosclerosis progression. These findings indicate that targeting this axis may offer a promising therapeutic strategy for the future treatment of atherosclerosis.

## Supplementary Material

Supplementary methods, figures and tables.

Supplementary excel 1.

## Abbreviations

AA: Aortic arch
ActD: Actinomycin D
AS: Atherosclerosis
Caspase1: Cysteine-aspartic acid protease 1
CHX: Cycloheximide
CL-Casp1: Cleaved Caspase 1
ECs: endothelial cells
GSDMD-N: N-terminal domain of Gasdermin D
HUVECs: Human umbilical vein endothelial cells
ICAM-1: Intercellular adhesion molecule 1
LMNA: Lamin A/C
LSS: Laminar shear stress
m6A: N6-methyladenosine
METTL14: Methyltransferase like 14
METTL14-N: the N-terminal domain of METTL14
NLRP3: NOD-like receptor thermal protein domain-associated protein 3
OSS: Oscillatory shear stress
PC: Partial ligation of the left carotid artery
RT-qPCR: Quantitative real-time PCR
TA: Thoracic aorta
VCAM-1: Vascular cell adhesion molecule 1
WTAP: WT1-associated protein
