# Supplementary material for: Mechanosensitive snoRNA-like circular RNA sno-circCNOT1 drives endothelial dysfunction and atherosclerosis
Source: Theranostics. 2026 Jan 1;16(6):2665–83. doi: 10.7150/thno.122995 (PMC12775674; doi:10.7150/thno.122995)
Supplement: Supplementary file 1 — Supplementary methods, figures and tables. [file thnov16p2665s1.pdf]

# Supplementary Materials

# Mechanosensitive snoRNA-like Circular RNA sno-circCNOT1 Drives Endothelial Dysfunction and Atherosclerosis

Lianru Bi[1,3#], Yihao Zhu[2,3#], Ziqi Chen[1,3#], Yiying Yang[1,3], Yanlong Leng[1], Huijie Wang[1,3], Jiajie Pan[1,3], Xiaozhe Zhang[1,3], Zekai Zeng[1,3], Yunjun Liang[3,5], Guifu Wu[1,3,4*] and Wendong Fan[2,3*]

*** Corresponding author:**

Wendong Fan (fanwd3@mail.sysu.edu.cn), PhD. Department of Cardiology, the First Affiliated Hospital of Sun Yat-sen University, No.58, Zhongshan 2nd Road, Guangzhou 510080, Guangdong Province, P.R. China.

Tel: +86 20 87330396; Fax: +86 20 87330396

Guifu Wu (wuguifu@mail.sysu.edu.cn), PhD. Department of Cardiology, the Eighth Affiliated Hospital of Sun Yat-sen University, No. 3025 Shennan Zhong Road, Shenzhen, 518033, Guangdong, P.R. China.

Tel: +86 0755-83982222; Fax: +86 0755-83980805

## This PDF file includes:

Materials and Methods

Supplementary Figures S1–S23

Supplementary Tables S1-S7

References 10,17

## Materials and Methods

### Cell culture

Human umbilical vein endothelial cells (HUVECs) and human aortic endothelial cells (HAECs) were maintained in endothelial cell medium (ScienCell, 1001) supplemented with 5% fetal bovine serum (FBS; Gibco). LentiX-293T cells (Clontech, 632180) were cultured in high-glucose Dulbecco's Modified Eagle Medium (DMEM; Gibco) containing 10% FBS. THP-1 cells (China National Collection of Authenticated Cell Cultures, Shanghai) were maintained in RPMI-1640 medium (Gibco, C11875500BT) containing 10% FBS. All cell cultures were kept at 37°C in a humidified incubator with 5% $CO_2$.

### Shear stress experiments

A shear stress system (Naturethink, Shanghai, China; NK110-STD) was employed to apply controlled shear stress, as previously described.[10] The system consisted of a peristaltic pump, parallel-plate flow chamber, unidirectional flow controller, medium reservoir, and silicone tubing (16#, 3.2 mm × 1.6 mm; LONGER). HUVECs or HAECs were seeded onto microscope slides and cultured to confluence. Cells were then exposed to either laminar shear stress (LSS; 15 dynes/cm$^2$) or oscillatory shear stress (OSS; 0.5 ± 4 dynes/cm$^2$). The flow system was kept at 37 °C in a humidified incubator containing 5% $CO_2$.

### Plasmid Construction, Lentivirus Production, and siRNA Transfection

The full-length human sno-circCNOT1 sequence was amplified and cloned into the pLO5-ciR vector (Geneseed, Guangzhou, China) to generate the sno-circCNOT1 overexpression plasmid. Additional constructs, including the sno-circCNOT1-PD, sno-circCNOT1-MS2-PD1, sno-circCNOT1-MS2-PD1, Sno-dele, Intron-Dele, and Exon-Dele, were similarly generated. For LMNA and METTL14 overexpression, the coding sequences of LMNA and METTL14 were inserted into the pLV-mCherry backbone (#36084; Addgene) to produce pLV-LMNA and pLV-METTL14, respectively. The METTL14 mutant (METTL14-MUT) was generated by polymerase chain reaction (PCR), replacing the native sequence in pLV-mCherry to create pLV-METTL14-MUT. Using similar strategy, HA-GFP-SNO-CTRL and HA-GFP-SNORA50A plasmids were constructed. The 3′ untranslated region (3′UTR) of NLRP3, carrying either the wild-type sequence or m$^6$A sites mutant (A-to-T substitutions), was inserted into the psiCHECK-2 vector (Promega, C8021).

All plasmids were validated by Sanger sequencing, and the corresponding primer sequences are listed in the Table S4.

For lentiviral production, LentiX-293T cells were transfected with the target plasmids together with psPAX2 (Addgene, #12260) and pMD2.G (Addgene, #12259) using the Lipo293 reagent (Beyotime Biotechnology, C0521). Viral supernatants were harvested and purified for subsequent experiments.

Small interfering RNAs (siRNAs; RIBOBIO, Guangzhou, China) were transfected into ECs using Lipofectamine 2000 (Thermo Fisher, 11668019), following the manufacturer's instructions. All siRNA sequences are provided in Table S1- S2.

**Quantitative reverse transcriptase polymerase chain reaction (RT-qPCR)**

RT-qPCR analysis was conducted according to our previous study. [17] Total RNA was extracted with TRIzol reagent (Sigma-Aldrich, T9424-200ML) following the manufacturer's guidelines. First-strand cDNA was generated from total RNA using the RevertAid First Strand cDNA Synthesis Kit (Thermo Fisher Scientific, K16225). RT-qPCR was conducted using LightCycler 480 SYBR Green I Master Mix (Roche, 4887352001-1) on a CFX96 Touch Real-Time PCR System (Bio-Rad, Berkeley, CA). Primers sequences are listed in Table S4.

**Endothelial Cell Treatment**

HUVECs or HAECs were treated for 24 h with one of the following: IL-1β (1 ng/mL; PeproTech, 200-01B-10), TNFα (5 ng/mL; Sino Biological, 10602-HNAE), oxidized LDL (ox-LDL) (50 μg/mL; Yiyuan Biotech, YB-002), or atorvastatin (ATV) (0.1 μM; Sigma-Aldrich, SML3030). Vehicle controls received matched volumes of solvent. After treatment, total RNA was isolated from the treated cells and analyzed by quantitative real-time PCR (RT-qPCR).

**Western blot analysis**

Cells or tissues were lysed in RIPA buffer (Thermo Fisher, 89900) containing protease and phosphatase inhibitors (Beyotime Biotechnology, P1045). Protein lysates were denatured in SDS-PAGE loading buffer (Beyotime Biotechnology, P0015), separated by SDS-PAGE, and transferred to polyvinylidene difluoride (PVDF) membranes (Millipore, IPVH00010). Following blocking in 5% BSA (Asegene, 43035-500) for 1 h at room temperature,

membranes were incubated with primary antibodies overnight at 4 °C. After washing, membranes were incubated with HRP-conjugated secondary antibodies for 1 h at room temperature. Protein signals were detected using ECL reagent (Millipore, WBKLS0100) according to the manufacturer's instructions. Band intensity quantification was performed using ImageJ software (NIH, Bethesda, MD, USA). Antibodies used are provided in Table S5.

**RNase R digestion assay**

Two micrograms of total RNA were incubated with RNase R (3U/μg; Beyotime Biotechnology, R7092S) at 37 °C for 15 minutes. Reactions were terminated by heat inactivation at 65 °C for 10 minutes. Untreated RNA samples served as negative controls. RNA was subsequently purified using TRIzol reagent. Divergent primers spanning the back-splice junction were designed to detect sno-circCNOT1, while convergent primers targeting exonic regions were used to quantify linear CNOT1 mRNA. Residual levels of sno-circCNOT1 and CNOT1 mRNA were measured by RT-qPCR.

**Actinomycin D assay**

Cells were treated with 5 ng/mL Actinomycin D (Sigma-Aldrich, A9415-2MG) for the indicated durations. Total RNA was then extracted, and the expression levels of sno-circCNOT1 and CNOT1 mRNA were analyzed by RT-qPCR.

**Nuclear and cytoplasmic RNA extraction**

Nuclear and cytoplasmic fractions from endothelial cells were isolated using the PARIS™ Kit (Thermo Fisher Scientific, AM1921) following the manufacturer's protocol. The expression level of sno-circCNOT1 in each fraction was quantified by RT-qPCR, with GAPDH and circ-CDR1-AS serving as cytoplasmic controls, and MALAT1 as the nuclear control.

**Fluorescence in situ hybridization (FISH)**

The intracellular distribution of sno-circCNOT1 was determined using the RNASweAMI™ FISH Kit (Servicebio, GF010-50T) according to the manufacturer's instructions. A Cy3-labeled probe specifically targeting the sno-circCNOT1 junction (designed and synthesized by Servicebio, Wuhan, China) was hybridized. Probe sequences are provided in Tables S3.

**RNA pull-down and Mass Spectrometry**

Endothelial cells were transfected with lentivirus encoding sno-circCNOT1-PD (control), sno-circCNOT1-MS2-PD1, or sno-circCNOT1-MS2-PD1 for 72 h. Cells were lysed in IP lysis buffer (Thermo Fisher Scientific, 87787). Lysates were incubated with GSH magnetic beads (Sigma-Aldrich, G0924-1ML) overnight at 4 °C with rotation. Proteins bound to RNA were eluted and separated using SDS-PAGE, following by detection through silver staining (Thermo Scientific, 24612). Selected protein bands were excised for mass spectrometry analysis（ProtTech, Inc. Suzhou, China).

**Immunofluorescence staining**

After fixed with 4% paraformaldehyde (15 min, room temperature), cells or tissue sections were permeabilized using 0.1% Triton X-100 and blocked for 1 h with 5% normal goat serum (NGS; BOSTER, AR0009). Primary antibodies were applied overnight at 4 °C, followed by secondary antibodies incubation for 1 h at room temperature. Nuclei were stained with DAPI (Abcam, ab104139-20ML), and images were acquired using confocal microscopy（LSM 800, Carl Zeiss, Germany).

**En face fluorescence staining**

Following anesthesia, mouse hearts were exposed via thoracotomy. A 26G needle was inserted into the left ventricular apex to sequentially perfuse heparinized saline and 4% paraformaldehyde for fixation. Isolated aortas were washed three times with 2.5% TBS-T (TBS [AR0031, BOSTER], 40 mL; Tween-20, 1 mL). Tissues samples were blocked in 5% normal goat serum (NGS), prepared in 2.5% TBS-T for 1 hour at room temperature with gentle agitation. Following this, samples were incubated overnight at 4 °C with primary antibodies diluted in 5% NGS. After three washes with 2.5% TBS-T, samples were incubated with fluorescent secondary antibodies for 1 hour at room temperature in the dark. Following three final washes with 2.5% TBS-T, tissues were counterstained with DAPI (Abcam, ab104139), mounted, and imaged using a laser scanning confocal microscopy（LSM 800, Carl Zeiss, Germany).

**Dual-luciferase reporter assay**

Luciferase activity was assessed 48 h after transfection in LentiX-293T cells using the Dual-Luciferase Assay Kit (Promega, E1910) following the manufacturer's protocol. Renilla

luciferase signals were normalized to Firefly luciferase for data analysis.

**Cell Adhesion Assay**

HUVECs were transfected with siRNAs targeting sno-circCNOT1, LMNA, or METTL14 or transduced with lentiviruses overexpressing these genes for 72 h. THP-1 cells ($5 \times 10^5$) were labeled with Calcein-AM (Thermo Fisher Scientific, C3100MP) following the manufacturer's protocol. After 30 min of incubation, labeled THP-1 cells were centrifuged (300 × g, 5 min), washed with PBS, and co-cultured with HUVECs at 37°C in the dark for 1 h. Non-adherent cells were removed by PBS washing, and adherent cells were observed under an inverted fluorescence microscope (Leica DMi8).

**Protein Stability Assay**

Cells were treated with 20 μM cycloheximide (Cell Signaling Technology, 2112S), 20 μM MG132 (Beyotime Biotechnology, S1748), or 0.1 μM bafilomycin A1 (MedChemExpress, HY-100558) for a specific time. Cells were harvested at each time point for subsequent Western blot analysis.

**Coimmunoprecipitation (Co-IP) assay**

HUVECs transduced with FLAG-tagged LMNA or METTL14 lentivirus for 72 h were lysed using IP lysis buffer (Thermo Fisher Scientific, 87787). Lysates were incubated overnight at 4 °C with anti-FLAG- magnetic beads (Beyotime Biotechnology, P2115). After washed, the bound proteins were eluted and analyzed by immunoblotting.

**RNA Immunoprecipitation assay**

Cells were lysed in IP lysis buffer (Thermo Fisher Scientific, 87787) on ice for 30 min. The obtained lysates were gently rotated overnight at 4 °C with anti-FLAG magnetic beads (Beyotime Biotechnology, P2115) to allow protein binding. RNA was extracted from beads, and sno-circCNOT1 levels were quantified by RT-qPCR.

**RNA-protein interaction simulation and analysis**

The full-length human LMNA structure predicted by AlphaFold was retrieved from UniProt. The three-dimensional structure of the RNA was built with 3dRNA. Protein–RNA docking was performed on the HDOCK server, with LMNA designated as the receptor and the RNA as the ligand (default parameters). Candidate complexes were ranked by HDOCK docking score (more negative values indicate better fits) and the confidence score (reliable when >0.7;

acceptable at 0.5–0.7). The top-ranked pose was visualized and annotated in PyMOL to illustrate the interaction interface.

**Partial ligation of carotid artery**

To induce disturbed flow-mediated atherosclerosis, a partial ligation of the left carotid artery was performed. Mice were anesthetized, and a 4–5 mm midline incision was made in the neck. The left carotid artery and its major branches were carefully exposed by blunt dissection. The superior thyroid artery was temporarily clamped. A small arteriotomy was created in the distal segment of the left external carotid artery using Vannas scissors, and a PE tube (outer diameter 0.32 mm, inner diameter 0.20 mm) was inserted 1–3 mm into the lumen. The opposite end of the tube was connected to a 32G (0.23 mm × 13 mm) syringe needle, and the tubing was secured to the artery with 7-0 sutures. The left common carotid artery was then transiently occluded proximal to the aortic arch, and AAV virus was slowly infused through the catheter. Following injection, vascular occlusion was maintained for 45 min to allow effective viral transduction within the arterial wall. After incubation, clamps on the common carotid artery and superior thyroid artery were released to restore blood flow. Hemostasis was confirmed, and the incision was closed. Mice were kept on a warming pad until full recovery and subsequently returned to their cages. All procedures were performed by the same operator to minimize technical variability.

**Histopathology**

Hematoxylin–eosin (H&E) staining: Carotid arteries were fixed in 4% paraformaldehyde, dehydrated through an ethanol gradient, embedded in paraffin, and sectioned at 5 μm thickness. The resulting sections underwent deparaffinization, rehydration, and Hematoxylin staining for 4 minutes, followed by counterstaining with eosin for 20 seconds.

Oil Red O staining: Fresh-frozen sections of carotid arteries were fixed in 4% paraformaldehyde for 15 minutes and stained with Oil Red O solution to visualize lipid deposits. Sections were afterwards counterstained with hematoxylin for 1 minute. Images were acquired using a digital slice scanner (NanoZoomer® S360), and lipid plaque area was quantified using Image J (NIH).

**Enzyme-linked immunosorbent assay (ELISA)**

Whole blood was subjected to clotting at room temperature for 2 hours, followed by

centrifugation at 3,000 ×g for 15 minutes. Serum concentrations of IL-1β, IL-18, and MCP-1 were determined with commercial ELISA kits in accordance with the manufacturer's instructions: IL-1β (Invitrogen, 88-7013), IL-18 (MULTISCIENCES, EK218) and MCP-1 (Servicebio, GEM0017).

**Serum lipid profile**

Following clot formation, serum separation was achieved by centrifugation at 3,000 × g for 15 minutes. Quantification of serum triglyceride (TG), total cholesterol (TC), and low-density lipoprotein cholesterol (LDL-C) levels was automatically performed using a biochemical analyzer (Chemray series, Rayto) following the manufacturer's standardized protocols.

**RNA Sequencing**

Total RNA was extracted from HUVECs transduced with sno-circCNOT1 lentivirus for 72 hours (n = 3) using TRIzol reagent (Invitrogen, CA, USA), following the manufacturer's guidelines. Assessment of RNA purity (A260/A280 ratio) and concentration was conducted using a NanoDrop 2000 spectrophotometer (Thermo Scientific, USA), while RNA integrity was evaluated with an Agilent 2100 Bioanalyzer (Agilent Technologies, Santa Clara, CA, USA). For library construction, the VAHTS Universal V6 RNA-seq Library Prep Kit (Vazyme, China) was employed, according to the manufacturer's instructions. Paired-end sequencing (150bp reads) was performed on an Illumina NovaSeq 6000 platform (IlluminaInc., SanDiego, CA, USA) by OE Biotech Co., Ltd. (Shanghai, China), followed by bioinformatic analysis including raw read alignment, differential expression quantification, and functional enrichment. The raw sequencing data are accessible at the NCBI Sequence Read Archive (SRA) database with the accession number PRJNA1246160.

**Circular RNA microarray Analysis**

Total RNA extraction was performed using TRIzol reagent, and ribosomal RNA (rRNA) depletion subsequently carried out using the Ribo-Zero™ rRNA Removal Kit. Digestion of linear RNA was conducted with RNase R (Epicentre, Inc.) and residual polyadenylated RNA was removed using poly(T)-conjugated magnetic beads. First-and second-strand cDNA synthesis was performed using random oligonucleotides and RNA templates. Library fragments were purified and size-selected using the AMPure XP system (Beckman Coulter, Beverly, CA, USA). Adapter-ligated DNA fragments were enriched using Illumina PCR

Primer Cocktail with 15 cycles of PCR. Purification and quantification of the final library products were conducted using the Agilent High Sensitivity DNA Assay on a Bioanalyzer 2100 system (Agilent Technologies, Santa Clara, CA, USA). Sequencing performed on Illumina NovaSeq 6000 platform by Shanghai Personal Biotechnology Co., Ltd. The raw sequencing data are accessible at the SRA with the accession number PRJNA1245494.

**Statistical analysis**

Data are expressed as the mean ± standard error of the mean (SEM) from at least three independent experiments. Statistical comparisons between two groups were analyzed using an unpaired two-tailed Student's *t* test. Multiple group comparisons were conducted using one-way or two-way analysis of variance (ANOVA) followed by Tukey's post hoc test. Statistical evaluations were performed in GraphPad Prism 9.5 (GraphPad Software), and $P < 0.05$ was considered statistically significant.

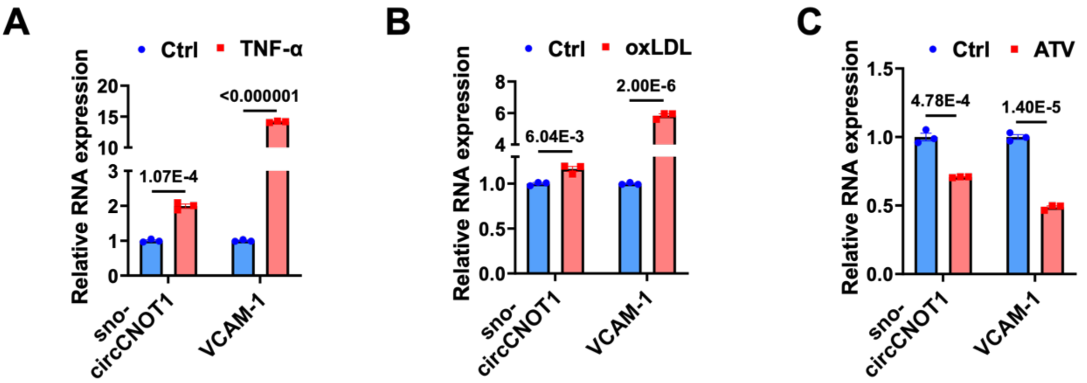


**Supplementary Figure S1. Modulation of sno-circCNOT1 by pro- and anti-atherogenic factors.** Endothelial cells were treated with TNFα (5 ng/mL; **A**), oxidized LDL (ox-LDL, 50 μg/mL; **B**), or atorvastatin (ATV, 0.1 μM; **C**) for 24 h. sno-circCNOT1 expression was quantified by RT-qPCR and normalized to GAPDH ($n$ = 3). Data are presented as mean SEM. Statistical significance was assessed using an unpaired two-tailed Student's t-test, with $P$ values reported.

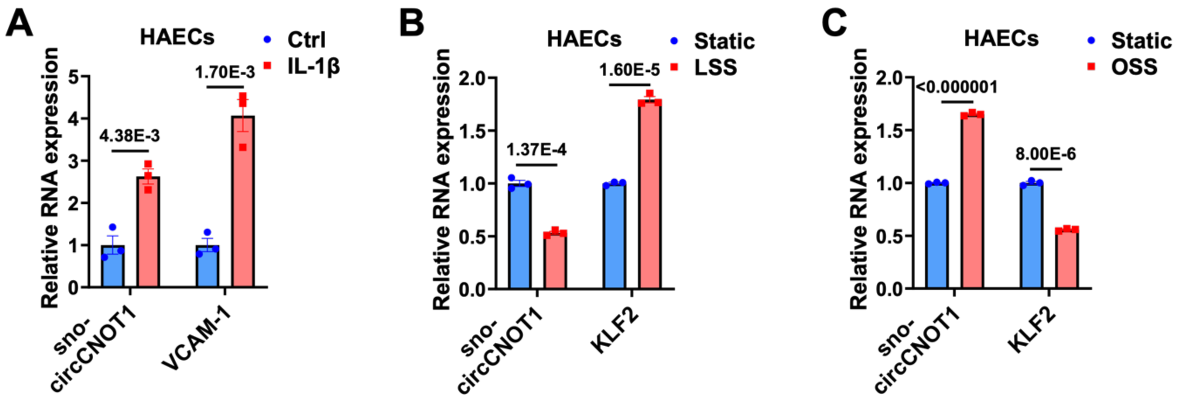


**Supplementary Figure S2. Pro-atherosclerotic IL-1β and oscillatory shear stress (OSS) up-regulate sno-circCNOT1 in human aortic endothelial cells (HAECs), whereas anti-atherosclerotic laminar shear stress (LSS) down-regulates its expression.** (**A**) RT-qPCR quantification of sno-circCNOT1 in HAECs treated with IL-1β; (**B**) exposed to LSS; or (**C**) exposed to OSS; normalized to GAPDH ($n$ = 3). VCAM-1 (IL-1β control) and shear-responsive KLF2 (LSS/OSS control) served as validation controls. Data are presented as mean SEM. Statistical significance was assessed using an unpaired two-tailed Student's t-test, with $P$ values reported.

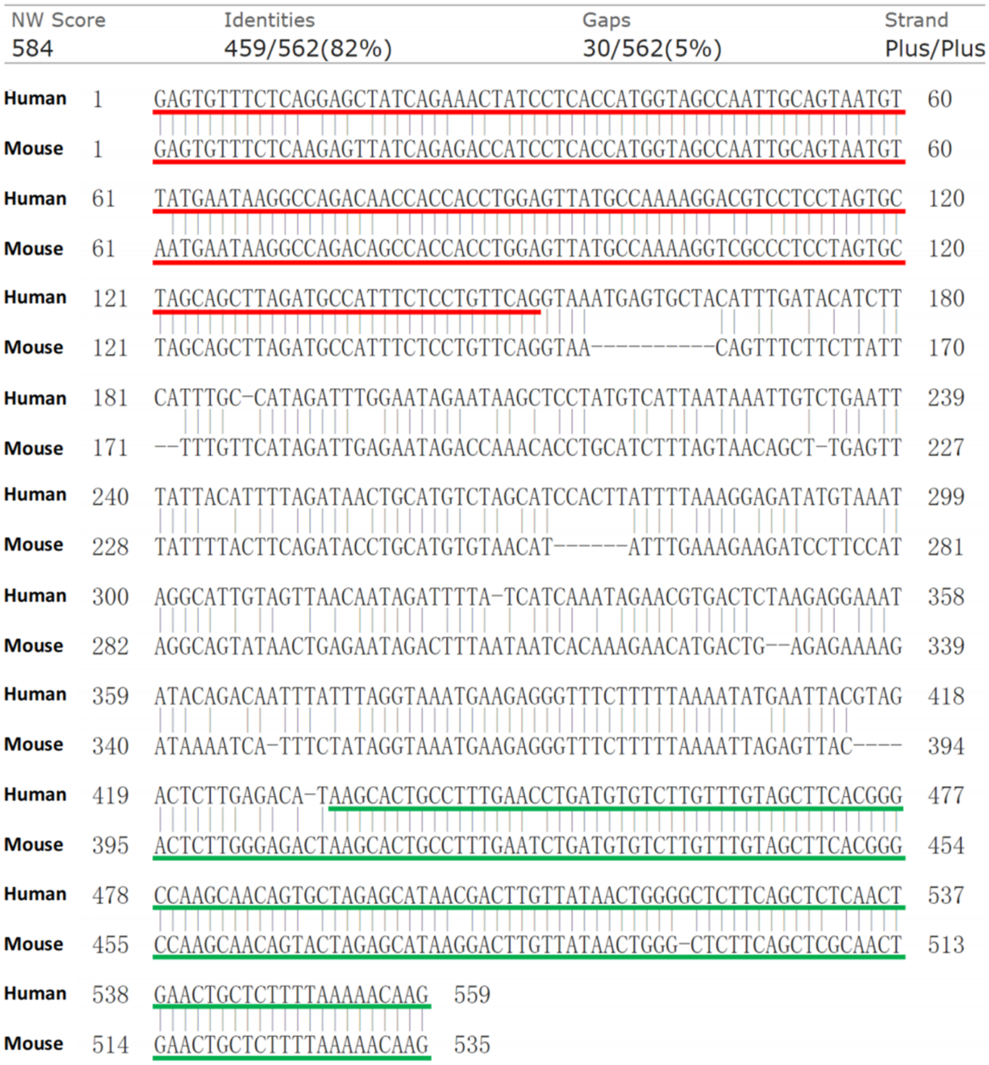


**Supplementary Figure S3. Genomic alignment of human and mouse sno-circCNOT1 regions.** The alignment spans exon 17 (red underline) and the adjacent intron 17 sequence containing the embedded SNORA50A snoRNA (green underline). Unmarked portions represent additional intron 17 sequences.

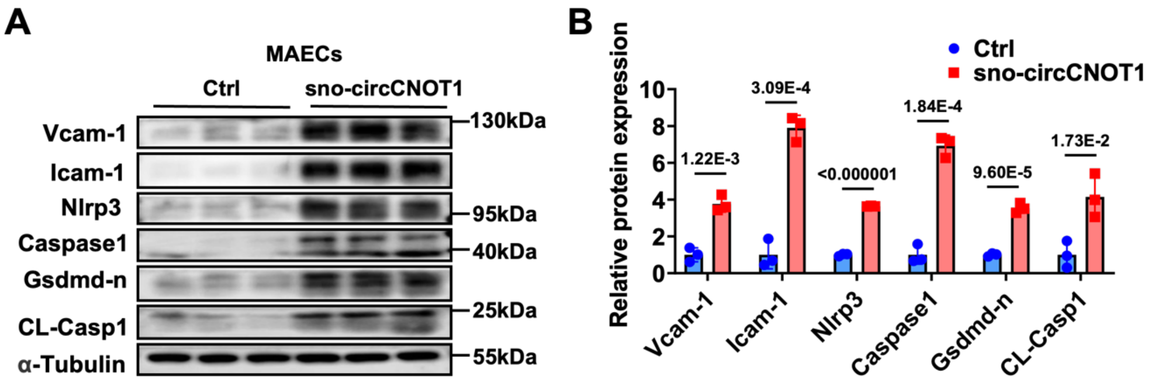


**Supplementary Figure S4. Human sno-circCNOT1 promotes pyroptosis and inflammation in mouse aortic endothelial cells (MAECs).** (**A**) MAECs were transduced with human sno-circCNOT1 or PLO5-ciR (control, Ctrl) lentivirus for 72 hours. Protein levels of Vcam-1, Icam-1, Nlrp3, Caspase-1, Gsdmd-n and CL-Casp1 were analyzed by western blot. (**B**) Quantification of proteins expression normalized to α-tubulin. n = 3. Data are presented as mean ± SEM. Statistical significance was assessed using an unpaired two-tailed Student's *t*-test, with *P* values reported.

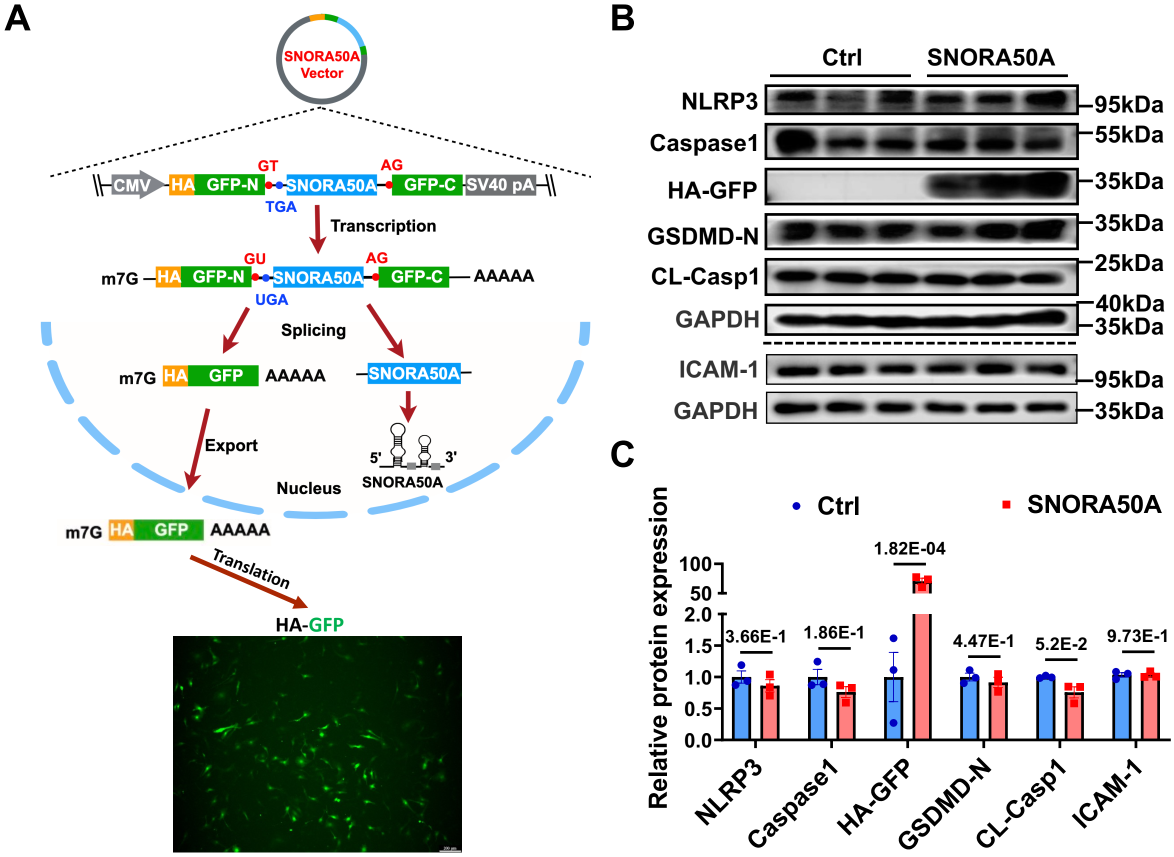


**Supplementary Figure S5. SNORA50A does not regulate endothelial pyroptosis or inflammation.** (**A**) Schematic of the snoRNA overexpression vectors. Green fluorescence protein (GFP) signals confirm successful snoRNA overexpression. Scale bar: 200 μm. Panel A was created using Figdraw. (**B-C**) HUVECs were transduced with SNORA50A or Ctrl lentivirus for 72 hours. Western blot analysis was performed to determine the protein levels of NLRP3, Caspase1, GSDMD-N, CL-Casp1 and ICAM-1. Quantification of protein expression normalized to GAPDH. Data are presented as mean ± SEM. Statistical significance was assessed using an unpaired two-tailed Student's *t*-test, with *P* values reported.

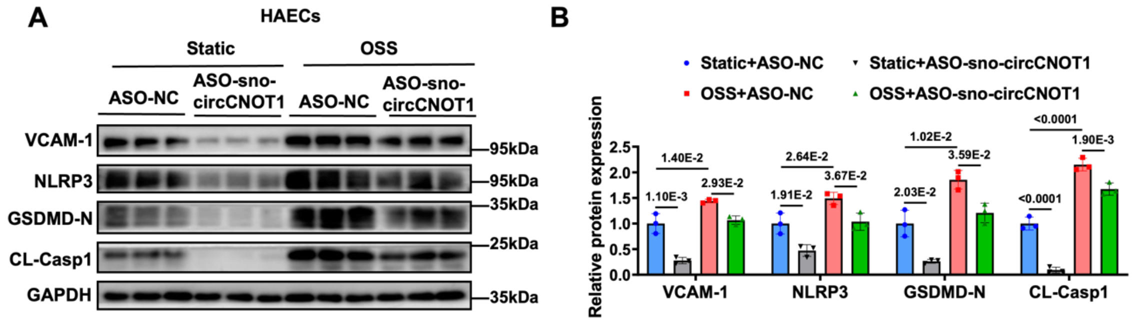


**Supplementary Figure S6. Knockdown of sno-circCNOT1 attenuates oscillatory shear stress (OSS)-induced upregulation of pyroptotic and inflammatory markers in primary human aortic endothelial cells (HAECs).** (**A–B**) Western blot analysis (**A**) and densitometric quantification (**B**) of the indicated proteins in HAECs transfected with control antisense oligonucleotide ASO-NC (Ctrl) or ASO-sno-circCNOT1, followed by OSS exposure (0.5 ± 4 dyn/cm², 1 Hz, 24 h; $n$ = 3). Data are shown as mean ± SEM. Statistical significance was assessed using a two-way analysis of variance (ANOVA) with Tukey's post hoc test, with $P$ values reported.

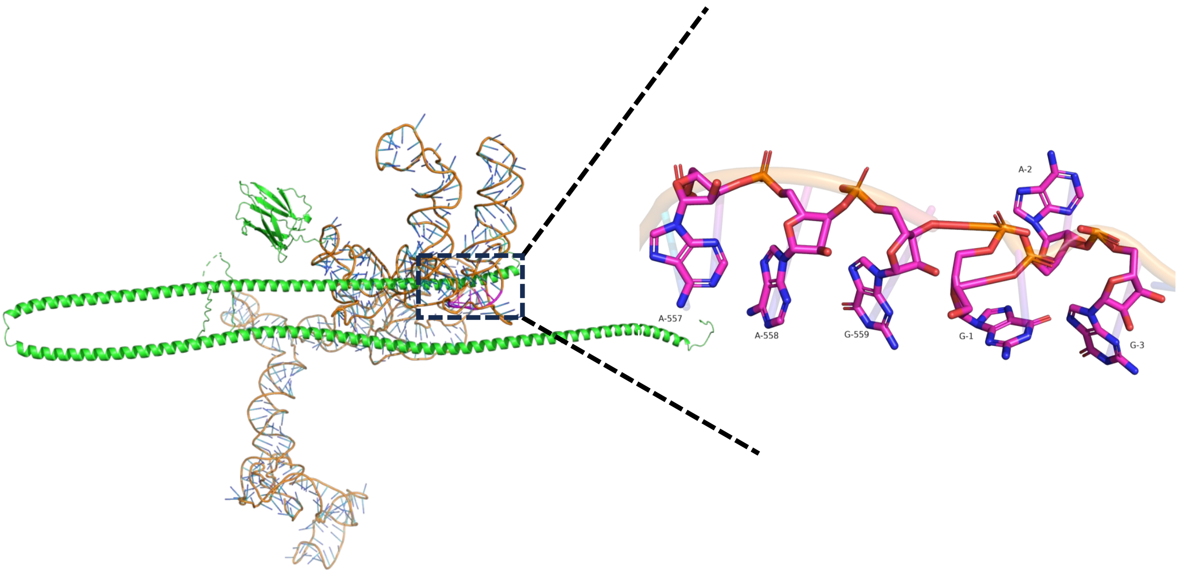


**Supplementary Figure S7. Computational model of the LMNA–sno-circCNOT1 interaction.** Full-length LMNA (green; AlphaFold) is docked to sno-circCNOT1 (orange/blue; 3dRNA), with the back-splice junction highlighted (magenta). HDOCK predicted a high-confidence binding pose (Docking score −218.83; Confidence 0.7984) where the LMNA intermediate filament rod (IF-ROD) domain interfaces with the sno-circCNOT1 splice-junction region. Visualization: PyMOL.

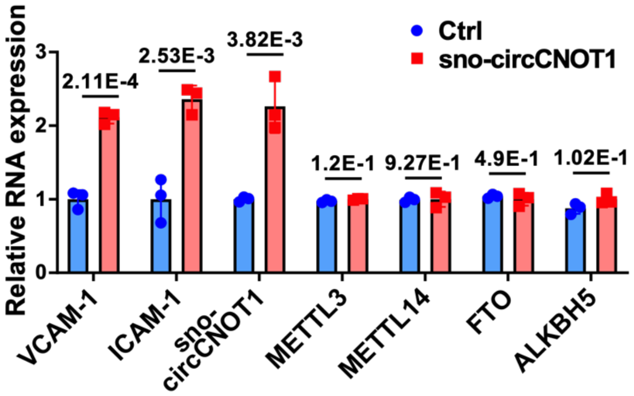


**Supplementary Figure S8. Sno-circCNOT1 does not alter mRNA levels of m6A regulators.** RNA levels of sno-circCNOT1, VCAM-1, ICAM-1, METTL3, METTL14, FTO and ALKBH5 in HUVECs transduced with control (Ctrl) or sno-circCNOT1 lentivirus were measured by RT-qPCR. GAPDH served as an internal reference. Data are presented as mean ± SEM. Statistical significance was assessed using an unpaired two-tailed Student's *t*-test, with *P* values reported.

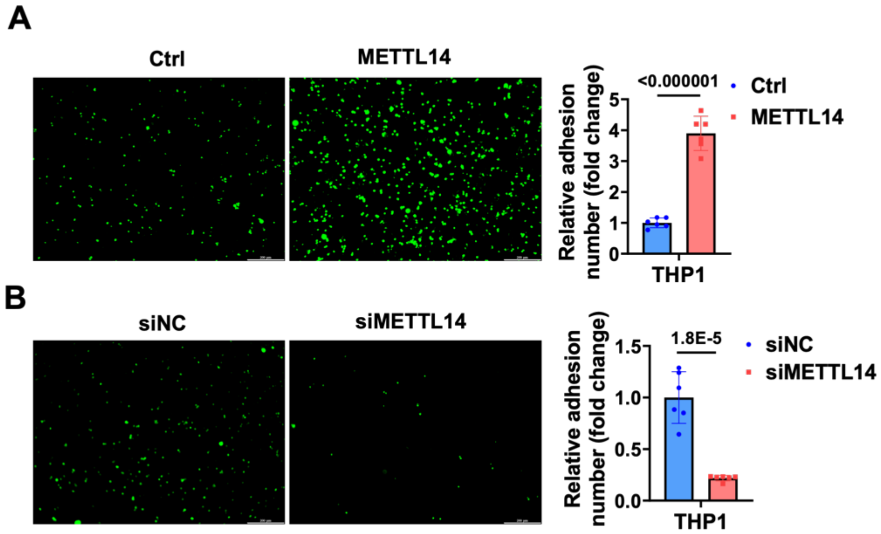


**Supplementary Figure S9. METTL14 promotes monocyte adhesion to endothelial cells.** Monocyte adhesion assays were performed by incubating METTL14-overexpressing (**A**) or METTL14-knockdown (**B**) HUVECs with fluorescently labeled THP-1 cells ($n$ = 6). Scale bar: 200 µm. Data are presented as mean ± SEM. Statistical significance was assessed using an unpaired two-tailed Student's *t*-test, with *P* values reported.

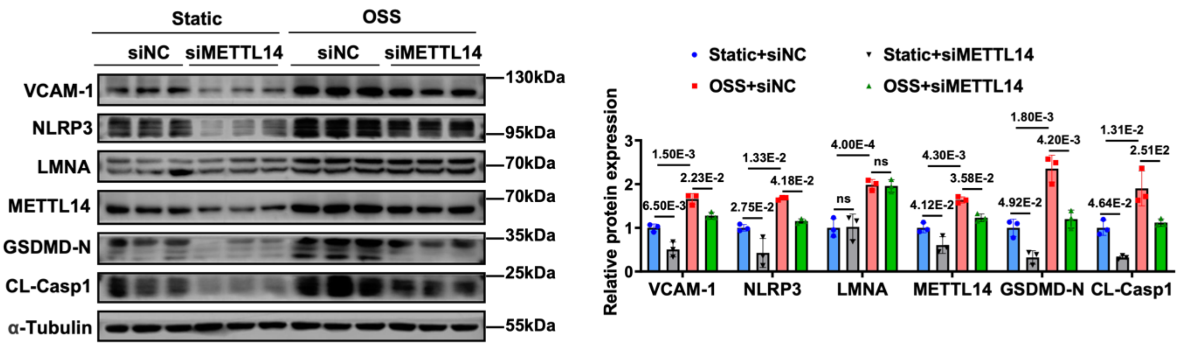


**Supplementary Figure S10. METTL14 knockdown reverses OSS-induced endothelial pyroptosis and inflammatory activation.** HUVECs were transduced with METTL14 siRNA (siMETTL14) or control siRNA (siNC) for 48 hours subsequently exposed to oscillatory shear stress (OSS) (0.5 ± 4 dyne/cm$^2$ at 1 Hz) for an additional 24 hours. Protein levels of VCAM-1, NLRP3, LMNA, METTL14, GSDMD-N and CL-Casp1 were analyzed by western blotting. Data are shown as mean ± SEM. Statistical significance was assessed using a two-way analysis of variance (ANOVA) with Tukey's post hoc test, with *P* values reported.

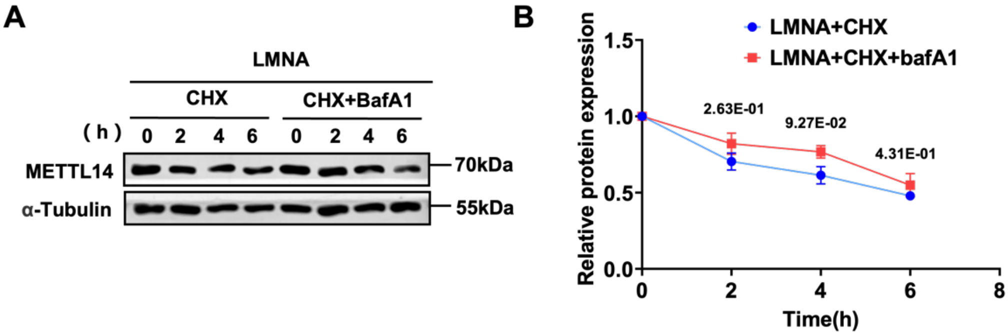


**Supplementary Figure S11. LMNA stabilizes METTL14 independently of the autophagy-lysosome pathway.** HUVECs transduced with LMNA-overexpressing lentivirus were treated with 0.1 μM lysosome inhibitor Bafilomycin A1 (BafA1) for the indicated durations. Western blot analysis was performed to determine the protein expression of METTL14 (*n* = 3). Data are presented as mean ± SEM. Statistical significance was assessed using an unpaired two-tailed Student's *t*-test, with *P* values reported.

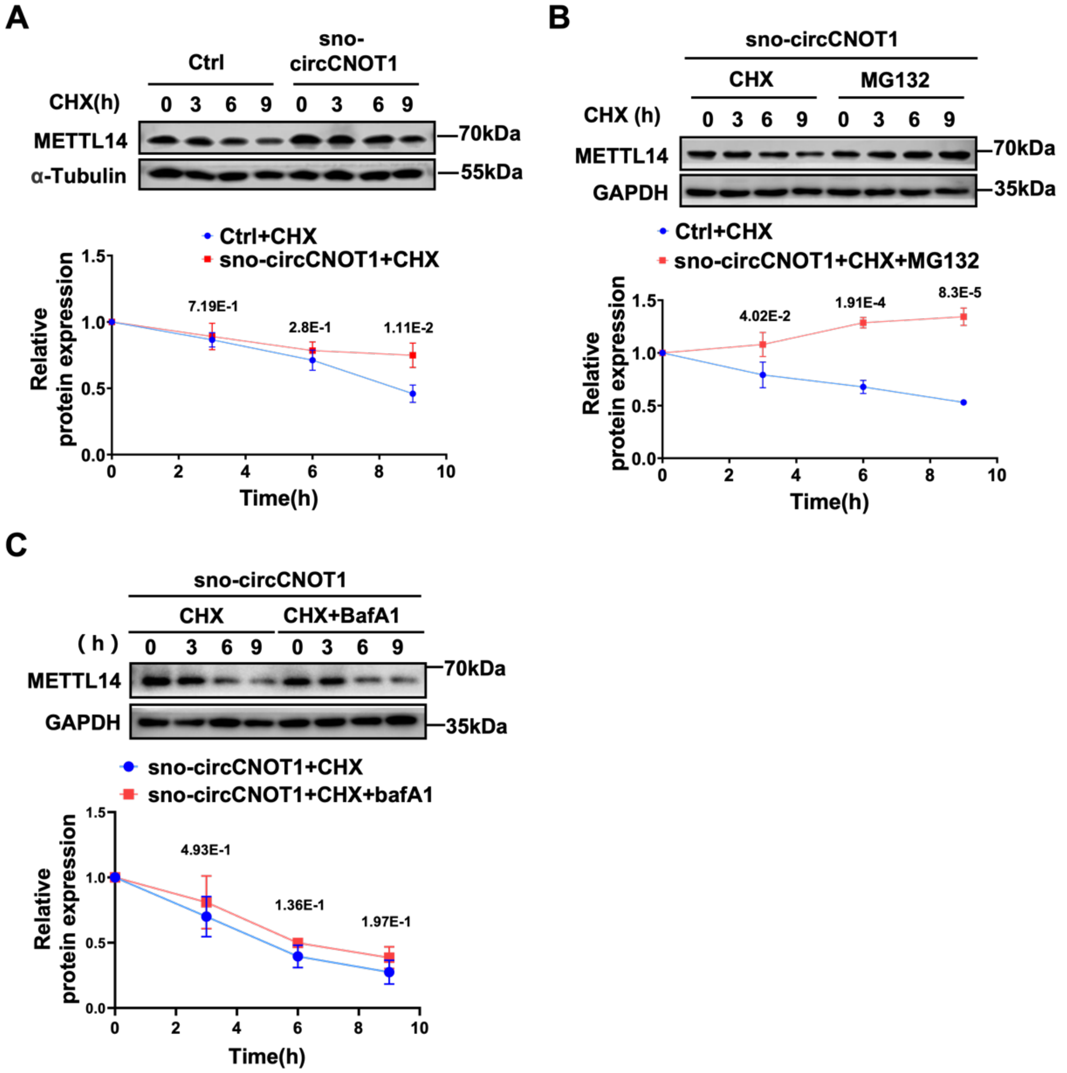


**Supplementary Figure S12. sno-circCNOT1 enhances METTL14 protein stability.** Western blot analysis of METTL14 protein levels in sno-circCNOT1-overexpressing HUVECs treated with: cycloheximide (CHX, 20μM) (**A**); CHX with or without proteasome inhibitor MG132 (20μM) (**B**); or CHX with or without 0.1 μM lysosome inhibitor BafA1 (**C**) for the indicated durations. Data are presented as mean ± SEM. Statistical analyses were assessed using an unpaired two-tailed Student's *t*-test, with *P* values reported.

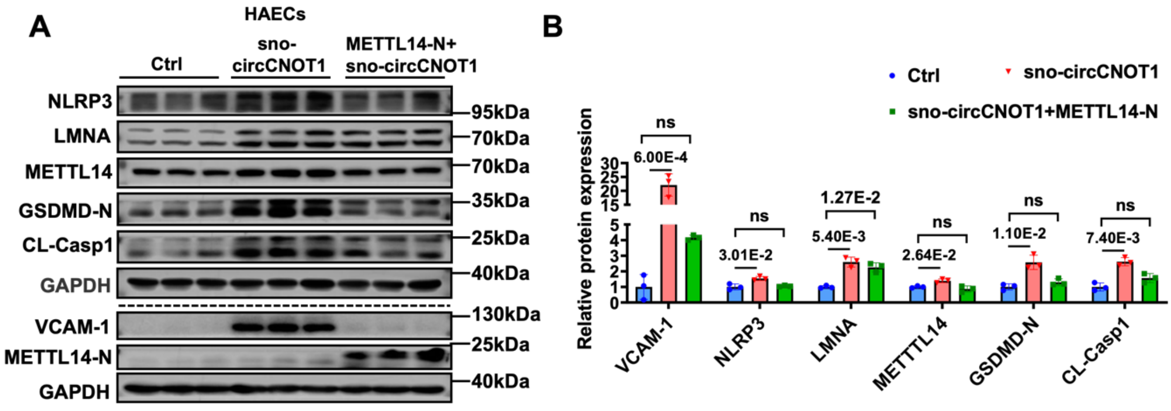


**Supplementary Figure S13. Overexpression of the N-terminal domain of METTL14 (METTL14-N) attenuated OSS-induced endothelial pyroptosis and inflammatory activation in human aortic endothelial cells (HAECs).** Western blot analysis (**A**) and densitometric quantification (**B**) of the indicated proteins in HAECs transduced for 72 h with either lentivirus expressing sno-circCNOT1 or lentiviruses co-expressing sno-circCNOT1 and METTL14-N. Data are shown as mean ± SEM. Statistical significance was assessed using a two-way analysis of variance (ANOVA) with Tukey's post hoc test, with *P* values reported.

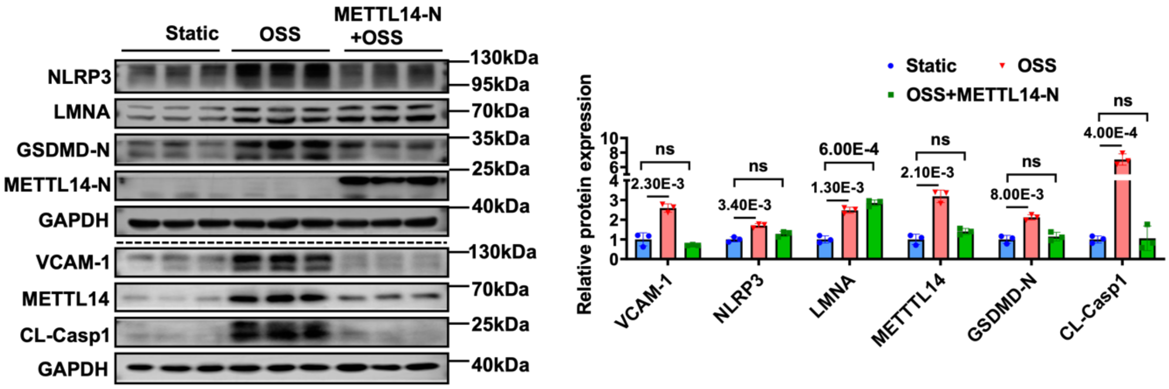


**Supplementary Figure S14. METTL14-N domain overexpression to disrupt the sno-circCNOT1/LMNA/METTL14 axis attenuates OSS-induced endothelial pyroptosis and inflammation.** HUVECs transduced with PLO5-ciR (Ctrl), or METTL14-N lentivirus for 48 hours were exposed to oscillatory shear stress (OSS; 0.5 ± 4 dyne/cm$^2$ at 1 Hz) or maintained under static (ST) conditions for an additional 24 hours. Western blotting assessed protein levels of VCAM-1, NLRP3, LMNA, METTL14, METTL14-N, GSDMD-N and cleaved caspase-1 (CL-Casp1) were analyzed by. Data are shown as mean ± SEM. Statistical significance was assessed using a two-way analysis of variance (ANOVA) with Tukey's post hoc test, with *P* values reported.

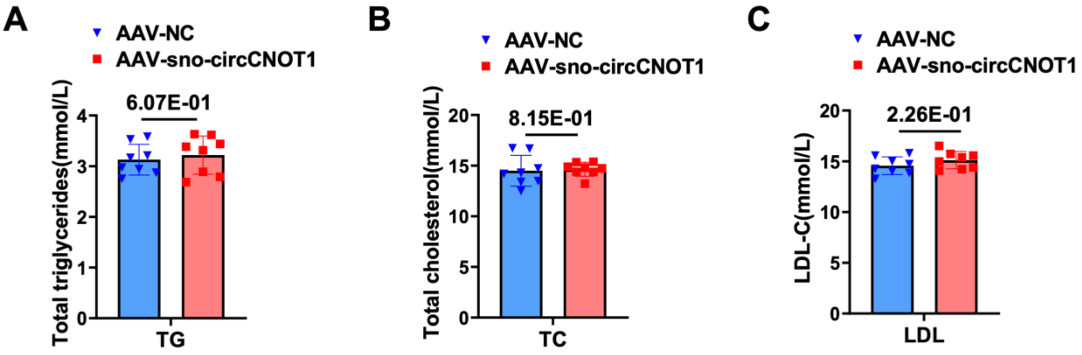


**Supplementary Figure S15. Serum lipid profiles are unaffected by AAV-sno-circCNOT1.** Serum levels of (**A**) triglycerides (TG), (**B**) total cholesterol (TC), and (**C**) low-density lipoprotein cholesterol (LDL-C) in mice treated with AAV-sno-circCNOT1 or AAV-NC control (n = 8). Statistical significance was assessed using a two-tailed Student's t-test. Data are presented as mean SEM, with *P* values reported.

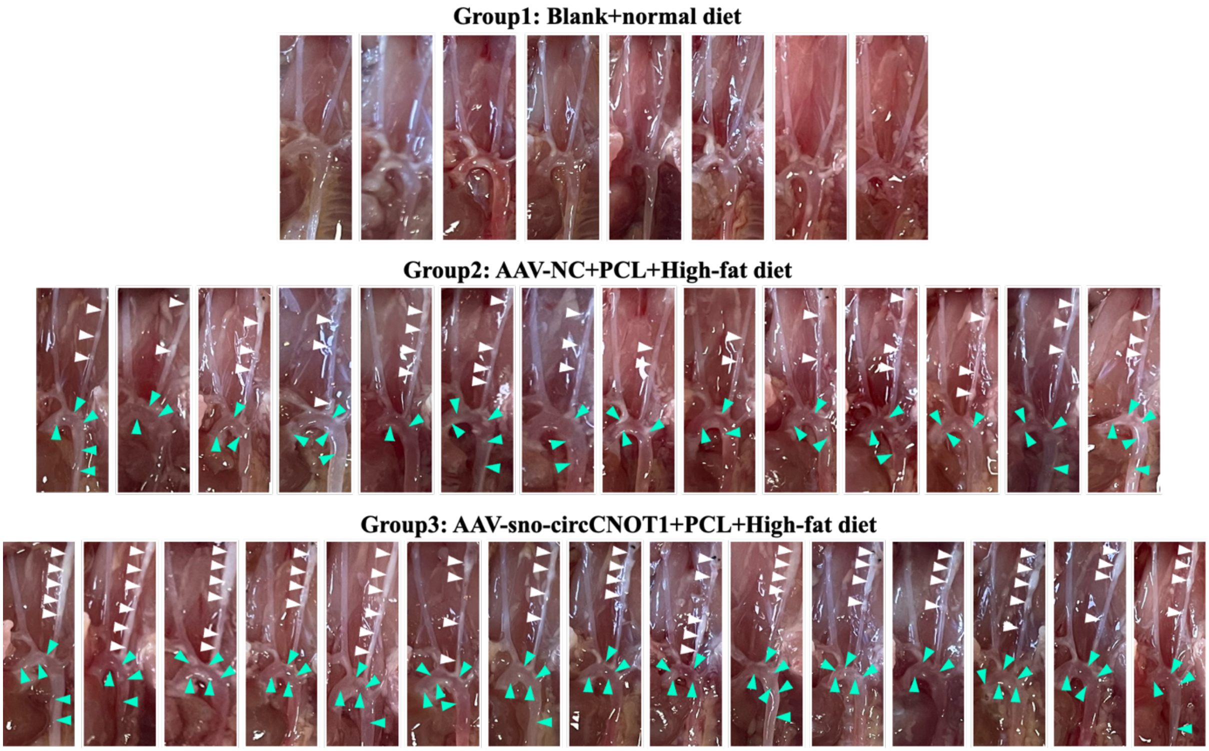


**Supplementary Figure S16. sno-circCNOT1 promotes atherosclerosis *in vivo*.** Atherosclerotic lesions in isolated carotid arteries and the aortic arch are shown. White arrows indicate plaque lesions in the left carotid artery (LCA); the green arrow indicates plaque lesions in the aortic arch (n = 8-15). PCL: partial left carotid artery ligation.

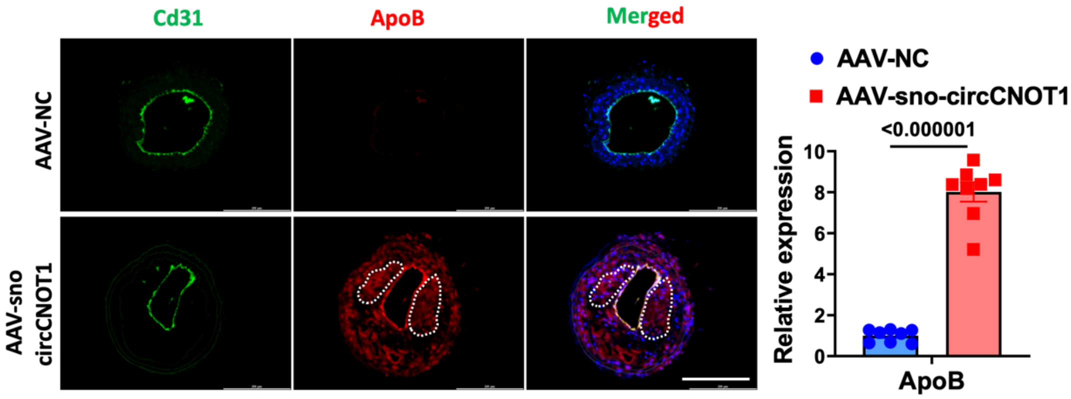


**Supplementary Figure S17. sno-circCNOT1 overexpression increases lipid deposition.** Representative immunofluorescence staining of ApoB (lesion lipid burden) and Cd31 (endothelial marker) in the left carotid artery sections; nuclei stained with DAPI (blue). Quantification data are shown on the right (Scale bar: 200 μm; n = 8). Region within dotted line indicates lipid core-like region. Statistical significance was assessed using an unpaired two-tailed Student's t-test. Data are presented as mean SEM, with *P* values reported.

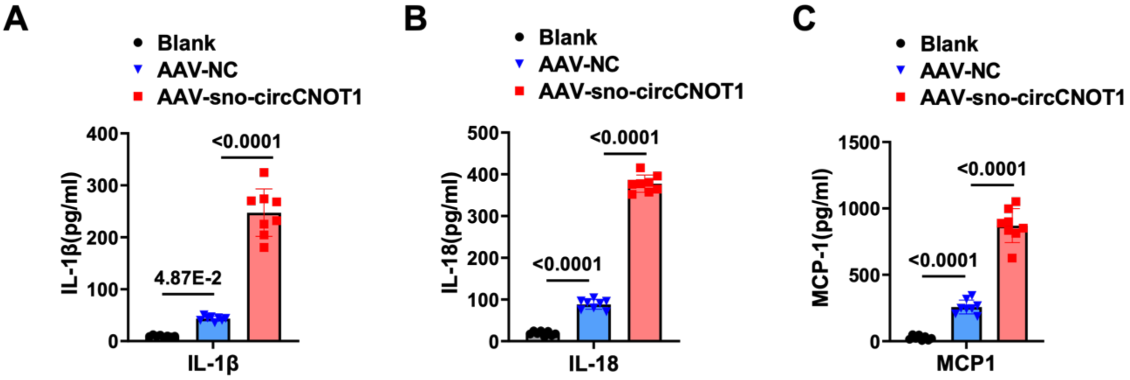


**Supplementary Figure S18. sno-circCNOT1 promotes atherosclerosis *in vivo*.** Plasma levels of interleukin (IL)-1β (**A**), IL-18 (**B**) and monocyte chemoattractant protein 1 (MCP1) (**C**) were detected by ELISA (n = 8). Data are presented as mean ± SEM. Statistical significance was assessed using an unpaired two-tailed Student's *t*-test, with *P* values reported.

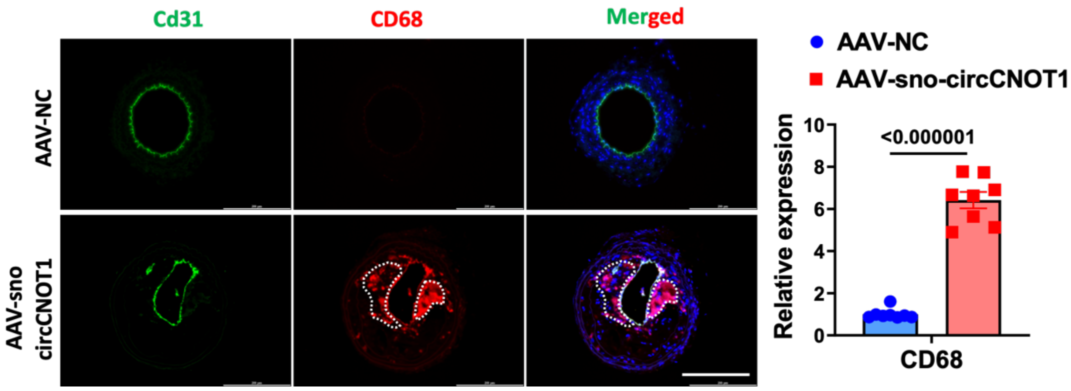


**Supplementary Figure S19. sno-circCNOT1 overexpression increases macrophage accumulation.** Representative immunofluorescence staining of CD68 (monocyte/ macrophage marker) and Cd31 (endothelial marker) in the left carotid artery sections; nuclei stained with DAPI (blue). Quantification data are shown on the right (Scale bar: 200 μm; n = 8). Region within dotted line indicates macrophage accumulation zone. The dotted-line regions in Supplementary Figures S17 and S19 illustrate partial colocalization of ApoB deposits with macrophage accumulation, indicating the initiation of a lipid-driven inflammatory loop that promotes atherosclerotic plaque progression. Statistical significance was assessed using an unpaired two-tailed Student's t-test. Data are presented as mean SEM, with *P* values reported.

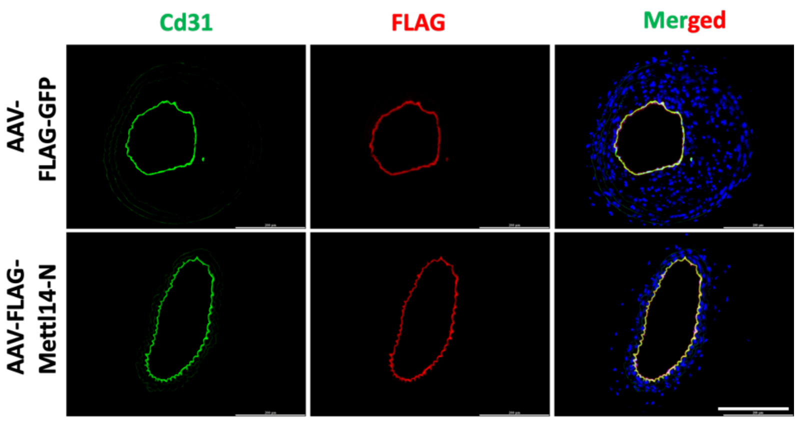


**supplementary Figure S20. AAV-FLAG-METTL14-N achieved efficient and specific endothelial expression in mice.** Representative immunofluorescence staining showing endothelial overexpression of FLAG (red) in the LCA. CD31 (green) marks endothelial cells. Scale bar: 200 μ m.

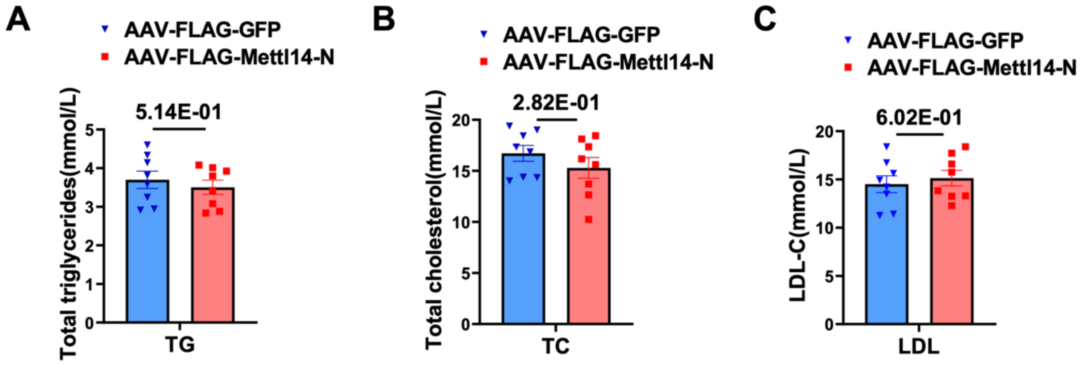


**Supplementary Figure S21. Serum lipid profiles are unaffected by AAV-FLAG-Mettl14-N.** Serum levels of (**A**) triglycerides (TG), (**B**) total cholesterol (TC), and (**C**) low-density lipoprotein cholesterol (LDL-C) in mice treated with AAV–FLAG-Mettl14-N or AAV-FLAG-GFP control (n = 8). Statistical significance was assessed using an unpaired two-tailed Student's t-test. Data are presented as mean SEM, with *P* values reported.

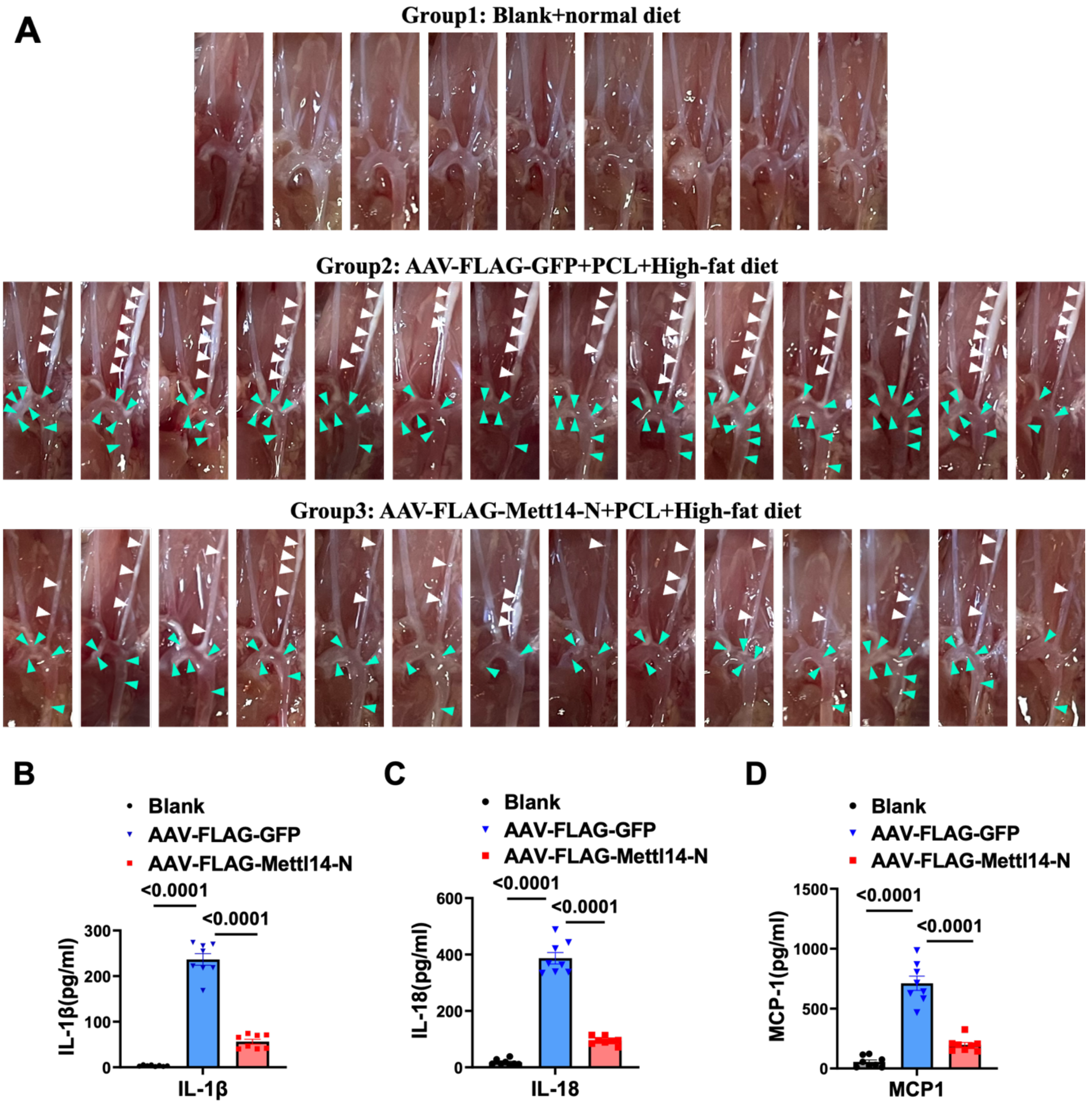


**Supplementary Figure S22. Mettl14-N inhibits atherosclerosis *in vivo*.** (**A**) Atherosclerotic lesions in isolated carotid arteries and the aortic arch are shown. White arrows indicate plaque lesions in the left common carotid artery (LCA); the green arrow indicates plaque lesions in the aortic arch (n = 8-15). PCL: partial left carotid artery ligation. (**B to D**) Plasma levels of IL-1β, IL-18 and monocyte chemoattractant protein 1 (MCP1) were detected by ELISA (n = 8). Data are presented as mean ± SEM. Statistical significance was assessed using an unpaired two-tailed Student's *t*-test, with *P* values reported.

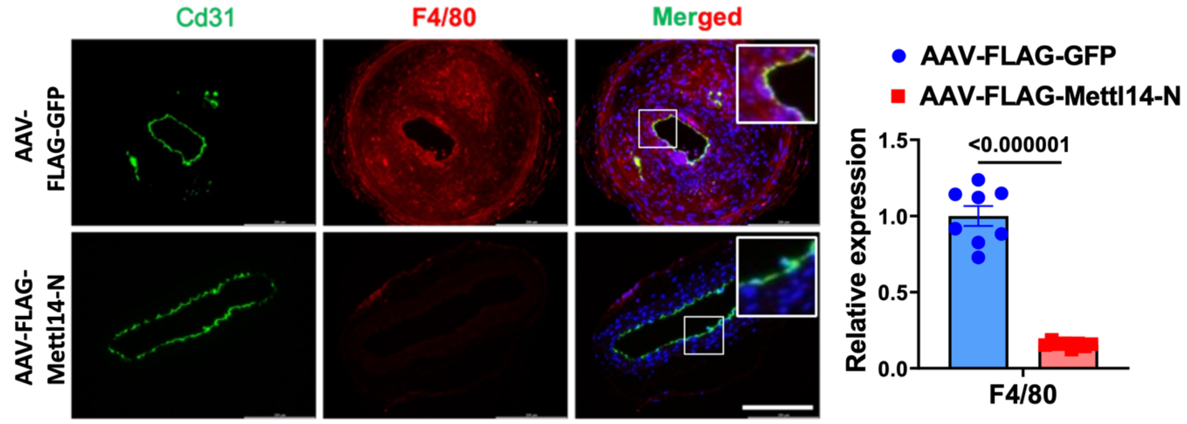


**Supplementary Figure S23. Mettl14-N inhibits atherosclerosis *in vivo*.** Representative immunofluorescence staining of F4/80 (macrophage marker) and Cd31 (endothelial marker) in ligated LCA sections. Nuclei were counterstained with DAPI (blue). Quantification data are shown on the right. Scale bar: 200 μm. $n$ = 8. Statistical significance was assessed using an unpaired two-tailed Student's t-test. Data are presented as mean SEM, with $P$ values reported.

## Supplementary Tables

**Supplementary Table S1. siRNAs sequences**

| Name | Sense Strand (5' -3') | Antisense Strand (5' -3') |
|---|---|---|
| LMNA siRNA | A.G.G.A.C.C.A.G.G.U.G.G. A.G.C.A.G.U.A. dTdT | U.A.C.U.G.C.U.C.C.A.C.C.U.G.G. U.C.C.U. dTdT |
| Control siRNA | U.C.U.C.U.C.U.U.C.G.C.G. C.A.C.C.U.A.U.dTdT | A.U.A.G.G.U.G.C.G.C.G.A.A.G.A. G.A.G.A.dTdT |
| METTL14 siRNA | G.C.A.A.A.G.A.U.G.A.G.C. A.G.AG.A.G.A. dTdT | U.C.U.C.U.C.U.G.C.U.C.A.U.C.U. U.U.G.C. dTdT |
| NLRP3 siRNA | C.G.U.A.A.G.A.A.G.U.A.C. A.G.A.A.A.G.U. dTdT | A.C.U.U.U.C.U.G.U.A.C.U.U.C.U. U.A.C.G. dTdT |

**Supplementary Table S2. ASO-sno-circCNOT1 target sequences**

| Name | sequences |
|---|---|
| ASO-sno-circCNOT1 **target sequences** | AACAAGGAGUGUUUCUCAGG |

**Supplementary Table S3. sno-circCNOT1 probe sequences**

| Name | sequences (5' -3') |
|---|---|
| sno-circCNOT1 | 5'-Cy3-CTGAGAAACACTCCTTGTTTTAAAAGAGC-CY3-3' |

**Supplementary Table S4. Primers**

| Name | Sense Primer (5' -3') | Antisense Primer (5' -3') |
|---|---|---|
| **Primers used to construct plasmids** | | |
| 3XFLAG-V | AATCCAGAGGTTGATTGT | GACCTCCATAGAAGACACCGACTC |

| | | |
|---|---|---|
| ector | CGACTTAGGATCCGCCGC CTCCACCCTTA | |
| 3XFLAG-LMNA | ATCCAGAGGTTGATTGTC GACTTACATGATGCTGCA GTTCTGGGGGCT | GACACCGACTCTAGAGCCGCCACCA TGGCTAGcGATTACAAAGACGATGA CGA |
| 3XFLAG-IF-ROD | TAATCCAGAGGTTGATTG TCGACTTACTCCAGTTTGC GCTTTTTGGT | GACCTCCATAGAAGACACCGACTC |
| 3XFLAG-LTD | TAATCCAGAGGTTGATTG TCGACTTAGCGCACCAGC TTGCGCA | TGGAGGCGGCGGATCCAAAAAGCG CAAACTGGAGTCCA |
| 3XFLAG-C-Terminal | TGGAGGCGGCGGATCCTC AGTGACTGTGGTTGAGGA CGA | ATCCAGAGGTTGATTGTCGACTTAC ATGATGCTGCAGTTCTGGGGGCT |
| 3XFLAG-IF-ROD-Del | ATCCAGAGGTTGATTGTC GACTTACATGATGCTGCA GTTCTGGGGGCT | TggAggCggcGGATCCAAAAAGCGCAA ACTGGAGTCCA |
| 3XFLAG-LTD-Del | ATCCAGAGGTTGATTGTC GACTTACATGATGCTGCA GTTCTGGGGGCT | GACCTCCATAGAAGACACCGACTC |
| 3XFLAG-C-Terminal-Del | TAATCCAGAGGTTGATTG TCGACTTAGCGCACCAGC TTGCGCA | GACCTCCATAGAAGACACCGACTC |
| pLO5-ciR | GACATTAATATTTCTTCTT TCGAATTCTAATACTTTCA G | GTATGGAGTTGTTAGCTAGGATCCA GT |

| | | |
|---|---|---|
| sno-circCNOT1 | TAATATTTCTTCTTTCGAATTCTAATACTTTCAGGAGTGTTTCTCAGGAGCTATCAGA | TGGAGTTGTTAGCTAGGATCCAGTTGTTCTTACCTTGTTTTAAAAGAGCAGTTCAGTTGAG |
| sno-circCNOT1-PD | TCCAGAGGTTGATTGTCGACttaTTTTGGAGGATGGTCGCCACC | ATAGAAGACACCGACTCTAGAGCCGCCACCATGGCTTCAAACTTTACTC |
| sno-circCNOT1-MS2-PD1 | TCCAGAGGTTGATTGTCGACttaTTTTGGAGGATGGTCGCCACC | ATAGAAGACACCGACTCTAGAGCCGCCACCATGGCTTCAAACTTTACTC |
| sno-circCNOT1-MS2-PD2 | TCCAGAGGTTGATTGTCGACttaTTTTGGAGGATGGTCGCCACC | AATTCCCGGGCTCGAGCTTTGGACTTCTTTTGAGATGTCATTTTTTGTGAC |
| R-deletion | TAATATTTCTTCTTTCGAATTCTAATACTTTCAGGAGTGTTTCTCAGGAGCTATCAGA | AGATGCATGCACTAGTCTTGTTTTTAAAAGAGCAGTTCAGTTGAG |
| HA-Ubi | TAATCCAGAGGTTGATTGTCGACTCAACCACCACGAAGTCTCAACACA | AGACACCGACTCTAGAGCCACCATGTACCCATACGATGTTCCAGATTACGCTGGTGGAG |
| 3XFLAG-METTL14 | TGGAGGCGGCGGATCCATGGATAGCCGCTTGCAGGAG | TAATCCAGAGGTTGATTGTCGACTTATCGAGGTGGAAAGCCACCTCTG |
| 3XFLAG-METTL14-N-terminal | ATCCAGAGGTTGATTGTCGACTTATCCCTTAAGAAAAGTACTAGAATCTTTGTA | TGGAGGCGGCGGATCCATGGATAGCCGCTTGCAGGAG |

| | | |
|---|---|---|
| | AATCT | |
| 3XFLAG-METTL14-MTD | TAATCCAGAGGTTGATTGTCGACTTATCGGTCAGATTTAGATTTGGGAGGAGGC | TGGAGGCGGCGGATCCACACAGAGCTTAAATCCCCATAATGAT |
| 3XFLAG-METTL14-MUT | ACTGCCGCCACCGCCTCGA | CCTCCATAGAAGACACCGACTCT |
| psiCHECK2-NLRP3-WT | ATTCTAGGCGATCGCTCGAGTGAAGAAATTTAAGATGCACTTAGAGGACTATCCTCCC | AATTCCCGGGCTCGAGGGGATGCAGCCCTTCTGGGGAGGATAGTCCTCTAAGTGCATCT |
| psiCHECK2-NLRP3-mut | ATTCTAGGCGATCGCTCGAGTGAAGAAATTTAAGATGCACTTAGAGGCCTATCCTCCC | AATTCCCGGGCTCGAGGGGATGCAGCCCTTCTGGGGAGGATAGGCCTCTAAGTGCATCT |
| sno-circCNOT1-sno-del | TAATATTTCTTCTTTCGAATTCTAATACTTTCAGGAGTGTTTCTCAGGAGCTATCAGA | AGTTGTTAGCTAGGATCCAGTTGTTCTTACCTTGTTTTAAAAGAGCA |
| sno-circCNOT1-Exon-del | TAATATTTCTTCTTTCGAATTCTAATACTTTCAGGAGTGTTTCTCAGGTA | TGGAGTTGTTAGCTAGGATCCAGTTGTTCTTACCTTGTTTTAAAAGAGCAGTTCAGTTGAG |
| sno-circCNOT1-Intron-del | TAATATTTCTTCTTTCGAATTCTAATACTTTCAGGAGTGTTTCTCAGGAGCTATCAGA | TGGAGTTGTTAGCTAGGATCCAGTTGTTCTTACCTTGTTTTAAAAGAGCAGTTCAGTTGAG |
| HA-GFP-S | GTAATCCAGAGGTTGATT | CCCGAAGGTTATGTACAGGTAAGTC |

| | | |
|---|---|---|
| NOCTRL | GTCGACTCATTTGTAGAG CTCATCCATGCCATGT | TCGAGTGAAGCGCTTCTCTTTC |
| HA-GFP-S NORA50A | GTAATCCAGAGGTTGATT GTCGACTCATTTGTAGAG CTCATCCATGCCATGT | GCCCGAAGGTTATGTACAGGTAAGT CTCGAGTGAAGCGCTG |
| **Primers for DNA agarose gel electrophoresis** | | |
| sno-circCN OT1divergent primers | CAAATAGAACGTGACTCT AAGAGGA | GTGGATGCTAGACATGCAGTT |
| sno-circCN OT1convergent primers | ACATTTGATACATCTTCAT TTGCCA | AGTTGAGAGCTGAAGAGCCC |
| **Primers for RT-qPCR** | | |
| GAPDH | TCGGAGTCAACGGATTTG GT | TTCCCGTTCTCAGCCTTGAC |
| CNOT1 | GATGTGGCCCAGGACTTG AA | AAAAGGCTCCCCATGCTCTC |
| sno-circCN OT1 | ACAAGGAGTGTTTCTCAG GAGC | AATGTAGCACTCATTTACCTGAACA |
| sno-circCN OT1 linear | TGACCCTTAAGAGTTTTCT TCCTG | CCTCTTAGAGTCACGTTCTATTTGAT GA |
| circRNA-C DR1-AS | CCAACGTCTCCAGTGTGC TG | CTTGAAGTCGCTGGAAGACCC |
| MALAT1 | AGGTCTGTCTGTTCTGTTG GC | TACTCCAAGCATTGGGGAACA |

| | | |
|---|---|---|
| VCAM-1 | GGACCACATCTACGCTGACAA | CTCCAGAGGGCCACTCAAAT |
| ICAM-1 | GGTAGCAGCCGCAGTCATAA | GATAGGTTCAGGGAGGCGTG |
| CCL2 | TAGCAGCCACCTTCATTCCC | GAACCCACTTCTGCTTGGGG |
| SELE | GCTCCAGGTGAACCCAACAA | AAACCAGGCTTCCATGCTCA |
| NLRP3 | GAGGCTGGCATCTGGATGAG | GTGTGTCCTGAGCCATGGAA |
| Caspase-1 | TCGCTTTCTGCTCTTCCACA | TCTTCACTTCCTGCCCACAG |
| GSDMD | CCTGCGTCAGGTTGCAGTT | GTCACAGGGATGAACTCCCC |
| LMNA | CACCGAGTCTGAAGAGGTGG | CTTCTTGGTATTGCGCGCTT |
| METTL14 | AGGGGTTGGACCTTGGAAGA | GAAGTCCCCGTCTGTGCTAC |
| METTL3 | GCGAGTGCCAGGAGATAGTC | ACACTGCTTGGTTGGTGTCA |

**Supplementary Table S5. Antibodies**

| Target antigen | Vender or Source | Catalog # | Working concentration | Lot# (preferred but not required) | Persistent ID/ URL |
|---|---|---|---|---|---|
| VCAM-1 | Cell Signaling Technology | 13662S | WB: 1:1000 | | https://www.cellsignal.cn/products/primary-antibodies/vcam-1-e1e8x-rabbit-mab/13662 |
| ICAM-1 | Cell Signaling Technology | 67836S | WB: 1:1000 | | https://www.cellsignal.cn/products/primary-antibodies/cd54-icam-1-e3q9n-xp-rabbit-mab/67836 |
| NLRP3 | Proteintech | 27458-1-AP | WB: 1:1000 | | https://www.ptgcn.com/products/NLRP3-Antibody-27458-1-AP.htm |
| GSDMD N-Terminal | Affinity Biosciences | DF13758-100ul | WB: 1:1000 | | https://www.affbiotech.cn/goods-18193-DF13758-GSDMD_N_Terminal_Antibody_Mouse_specific_.html |
| Cleaved-Caspase 1 | Affinity Biosciences | AF4005-50u | WB: 1:1000 | | https://www.affbiotech.cn/goods-15750-AF4005-Cleaved_Caspase_1_Asp296_p20_Antibody.html |
| Caspase-1 | Cell Signaling Technology | 3866S | WB: 1:1000 | | https://www.cellsignal.cn/products/primary-antibodies/caspase-1-d7f10-rabbit-mab/3866 |
| Lamin A/C | Proteintech | 10298-1-AP-100ul | WB: 1:1000 | | https://www.ptgcn.com/products/lamin-A-Antibody-10298-1-AP.htm |
| eNOS | Cell | 5880S | WB: | | https://www.cellsignal |

| | | | | | |
|---|---|---|---|---|---|
| | Signaling Technology | | 1:1000 | | .cn/products/primary-antibodies/enos-6h2-mouse-mab/5880 |
| KLF2 | Bioss | bs-2772R-100ul | WB: 1:1000 | | http://www.biosschina.com/#/productDetail?goods_id=26440 |
| GAPDH | Proteintech | 60004-1-IG-1000UL | WB:0.1pg/mL | | https://www.ptgcn.com/products/GAPDH-Antibody-60004-1-Ig.htm |
| α-Tubulin | Proteintech | 66031-1-IG-500UL | WB: 1:20000 | | https://www.ptgcn.com/products/tubulin-Alpha-Antibody-66031-1-Ig.htm |
| HA-Tag | Cell Signaling Technology | 34888S-100 μl | WB: 1:1000 | | https://www.cellsignal.cn/products/antibody-conjugates/ha-tag-c29f4-rabbit-mab-alexa-fluor-350-conjugate/34888 |
| DYKDDDDK Tag | Cell Signaling Technology | 14793S | WB: 1:1000<br><br>IHC: 1:1000 | | https://www.cellsignal.cn/products/primary-antibodies/dykddddk-tag-d6w5b-rabbit-mab-binds-to-same-epitope-as-sigma-aldrich-anti-flag-m2-antibody/14793 |
| APOB | Proteintech | 20578-1-AP-50ul | IHC: 1:100 | | https://www.ptgcn.com/results?category=&filter=&q=20578-1-AP-50ul |
| CD68 | Abcam | ab303565-40 | IHC: 1:100 | | https://www.abcam.cn/products/primary-antibodies/cd68-antibody-rm1031-ab303565 |
| NLRP3 | Proteintech | 68102-1-Ig-100ul | IHC: 1:200 | | https://www.ptgcn.com/products/NLRP3-Antibody-68102-1-Ig.htm |
| GSDMD N-Terminal | Affinity Biosciences | DF13758-100ul | IHC: 1:200 | | https://www.affbiotech.cn/goods-18193-DF13758-GSDMD_N_Terminal_Antibody_M |

| | | | | | |
|---|---|---|---|---|---|
| | | | | | ouse_specific_.html |
| Cleaved-Caspase 1 | Affinity Biosciences | AF4005-50ul | IHC: 1:200 | | https://www.affbiotech.cn/goods-15750-AF4005-Cleaved_Caspase_1_Asp296_p20_Antibody.html |
| Lamin A/C | Cell Signaling Technology | 4777T | IHC: 1:200 | | https://www.cellsignal.cn/products/primary-antibodies/lamin-a-c-4c11-mouse-mab/4777 |
| METTL14 | Proteintech | 26158-1-AP-50UL | IHC: 1:100 | | https://www.ptgcn.com/products/METTL14-Antibody-26158-1-AP.htm |
| CD31 | Abcam | Ab182981 | IHC: 1:1000 | | https://www.abcam.cn/products/primary-antibodies/cd31-antibody-epr17259-ab182981.html |
| F4/80 | Proteintech | 29414-1-AP-50ul | IHC: 1:8000 | | https://www.ptgcn.com/products/F4-80-Antibody-29414-1-AP.htm |
| VCAM-1 | Abcam | ab134047-100 | IHC: 1:500 | | https://www.abcam.cn/products/primary-antibodies/vcam1-antibody-epr5047-ab134047.html |
| Goat anti-rat IgG H&L (Alexa Fluor® 594) | Abcam | ab150168 | IHC: 1:1000 | | https://www.abcam.cn/products/secondary-antibodies/goat-rat-igg-hl-alexa-fluor-594-preadsorbed-ab150168.html |
| Goat anti-mouse IgG H&L (Alexa Fluor® 594) | Abcam | ab150116 | IHC: 1:1000 | | https://www.abcam.cn/products/secondary-antibodies/goat-mouse-igg-hl-alexa-fluor-594-ab150116.html |
| Goat Anti-Rabbit IgG H&L | Abcam | ab150077 | IHC: 1:1000 | | https://www.abcam.cn/products/secondary-antibodies/goat-rabbit-i |

| | | | | | |
|---|---|---|---|---|---|
| (Alexa Fluor® 488) | | | | | gg-hl-alexa-fluor-488-ab150077.html |
| VE-Cadherin | Abcam | ab33168 | IHC: 1:400 | | https://www.abcam.cn/products/primary-antibodies/ve-cadherin-antibody-intercellular-junction-marker-ab33168.html |
| VE-Cadherin | Immunoway | YM3762-50ul | IHC: 1:400 | | https://www.immunoway.com/products/primary-antibodies/YM3762-VE-Cadherin-3G8-Mouse-mAb.html |

**Supplementary Table S6. Cultured Cells**

| **Name** | **Vendor or Source** | **Sex (F, M, or unknown)** | **Persistent ID / URL** |
|---|---|---|---|
| HUVECs | ScienCell | unknown | https: / / www. sciencellonline. com/ products-services/ primary- cells/ human/ cell- types/ endothelial - cells/ human- umbilical- vein- endothelial- cells. html |
| HAECs | iCell | unknown | https://www.icellbioscience.com/cellDetail/1507 |
| Lenti-X 293T | Clontech | unknown | https: / / www. takarabio. com/ products/ gene-function/ viral- transduction/ lentivirus/ packaging- systems-and-cells/lenti-x-293t-cells?catalog=632180 |
| THP- 1 | China National Collection of Authenticated Cell Cultures | unknown | https://www.cellbank.org.cn/search-detail.php?id=517 |
| MAECs | BeNa Culture Collection | unknown | https://www.bncc.com/pro/p1/1/p_359881.html |

**Supplementary Table S7. Overexpression Vectors**

| **Name** | **Vendor or Source** | **Catalog #** | **Lot # (preferred but not required)** |
|---|---|---|---|
| pLO5-ciR | Guangzhou Geneseed Biotech Co., Ltd | GS0107 | |
| AAV-ENT | Shanghai GeneChemCo., Ltd | AAVENT-CIRCRNA (73140-1) | AAV-ENT |
| PsPAX2 | addgene | Plasmid #12260 | PsPAX2 |
| pMD2.G | addgene | Plasmid #12259 | pMD2.G |

## References


10. Wang R, Zeng Y, Chen Z, Ma D, Zhang X, Wu G, Fan W. Shear-Sensitive circRNA-LONP2 Promotes Endothelial Inflammation and Atherosclerosis by Targeting NRF2/HO1 Signaling. *JACC Basic Transl Sci*. 2024;9:652-670. doi: 10.1016/j.jacbts.2024.02.019

17. Mo W, Chen Z, Zhang X, Dai G, Ma D, Pan J, Zhang X, Wu G, Fan W. N6-Methyladenosine Demethylase FTO (Fat Mass and Obesity-Associated Protein) as a Novel Mediator of Statin Effects in Human Endothelial Cells. *Arterioscler Thromb Vasc Biol*. 2022; 42:644-658. doi: 10.1161/atvbaha.121.317295
