# Supplementary material for: Mechanosensitive snoRNA-like circular RNA sno-circCNOT1 drives endothelial dysfunction and atherosclerosis
Source: Theranostics. 2026 Jan 1;16(6):2665–83. doi: 10.7150/thno.122995 (PMC12775674; doi:10.7150/thno.122995)
Supplement: Supplementary file 2 — Supplementary excel 1. [file thnov16p2665s2.pdf]

CHENZIQI_S1_032022

Hits	Protein Mass	No. of Peptide	Sequence Header	Link	Relative Abundance	Probability	Score	SI	AA No	SIn*1E6	No. of Unique Peptide
1	66096.9	14	>sp|Q07065|CKAP4_HUMAN Cytoskeleton-associated protein 4 OS=Homo sapiens GN=CKAP4 PE=1 SV=2	Q07065	16.4%	99.0%	209	60349	602.00	236.87415	13
2	74379.9	12	>sp|P02545|LMNA_HUMAN Prelamin-A/C OS=Homo sapiens GN=LMNA PE=1 SV=1	P02545	13.1%	99.0%	189	52936	664.00	188.37667	10
4	71244.3	11	>sp|P02769|ALBU_BOVIN Serum albumin OS=Bos taurus GN=ALB PE=1 SV=4	P02769	9.1%	99.0%	138	33690	607.00	131.14641	7
5	53676.2	9	>sp|P08670|VIME_HUMAN Vimentin OS=Homo sapiens GN=VIM PE=1	P08670	8.2%	99.0%	122	23386	466.00	118.58073	8
16	83554.3	2	>sp|P08238|HS90B_HUMAN Heat shock protein HSP 90-beta OS=Homo sapiens GN=HSP90AB1 PE=1 SV=4	P08238	7.0%	97.5%	23	30885	724.00	100.79828	2
6	76991.6	9	>sp|P0CG48|UBC_HUMAN Polyubiquitin-C OS=Homo sapiens GN=UBC PE=1 SV=3	P0CG48	6.9%	87.9%	126	28944	685.00	99.841721	1
7	71082.4	4	>sp|P11142|HSP7C_HUMAN Heat shock cognate 71 kDa protein OS=Homo sapiens GN=HSPA8 PE=1	P11142	4.6%	99.0%	57	18042	646.00	65.992752	4
3	227645	11	>sp|P35579|MYH9_HUMAN Myosin-9 OS=Homo sapiens GN=MYH9 PE=1 SV=4	P35579	4.5%	99.0%	173	53356	1960.00	64.323738	11
10	51229.5	4	>sp|P61978|HNRPK_HUMAN Heterogeneous nuclear ribonucleoprotein K OS=Homo sapiens GN=HNRNPK PE=1 SV=1	P61978	4.4%	99.0%	60	12399	463.00	63.277563	4
11	68641	3	>sp|P04843|RPN1_HUMAN Dolichyl-diphosphooligosaccharide--protein glycosyltransferase subunit 1 OS=Homo sapiens GN=RPN1 PE=1	P04843	3.8%	99.0%	39	14172	607.00	55.16791	3
14	42051.9	2	>sp|P60709|ACTB_HUMAN Actin, cytoplasmic 1 OS=Homo sapiens GN=ACTB PE=1 SV=1	P60709	3.7%	97.8%	25	8428	375.00	53.105235	2
26	48311.3	1	>sp|Q07666|KHDR1_HUMAN KH domain-containing, RNA-binding, signal transduction-associated protein 1 OS=Homo sapiens GN=KHDRBS1 PE=1 SV=1	Q07666	2.3%	91.9%	15	6262	443.00	33.400534	1
19	70019.7	2	>sp|Q03252|LMNB2_HUMAN Lamin-B2 OS=Homo sapiens GN=LMNB2 PE=1 SV=4	Q03252	2.3%	98.0%	24	8541	620.00	32.550758	2
12	69618	3	>sp|P17844|DDX5_HUMAN Probable ATP-dependent RNA helicase DDX5 OS=Homo sapiens GN=DDX5 PE=1 SV=1	P17844	1.9%	99.0%	36	7220	614.00	27.78516	3
9	133291	4	>sp|P07996|TSP1_HUMAN Thrombospondin-1 OS=Homo sapiens GN=THBS1 PE=1 SV=2	P07996	1.8%	99.0%	50	13196	1170.00	26.650203	4
18	60053.7	2	>sp|Q9Y2X3|NOP58_HUMAN Nucleolar protein 58 OS=Homo sapiens GN=NOP58 PE=1 SV=1	Q9Y2X3	1.6%	98.4%	26	5302	529.00	23.682532	2
17	61293.8	2	>sp|Q9NZN4|EHD2_HUMAN EH domain-containing protein 2 OS=Homo sapiens GN=EHD2 PE=1 SV=2	Q9NZN4	1.5%	96.7%	22	4880	543.00	21.235577	2
24	114322	1	>sp|O75150|BRE1B_HUMAN E3 ubiquitin-protein ligase BRE1B OS=Homo sapiens GN=RNF40 PE=1	O75150	1.2%	88.4%	13	7081	1001.00	16.714931	1
22	63897.8	1	>sp|P21589|5NTD_HUMAN 5"-nucleotidase OS=Homo sapiens GN=NT5E PE=1 SV=1	P21589	1.1%	86.1%	13	3962	574.00	16.309726	1
15	76167.8	2	>sp|P08133|ANXA6_HUMAN Annexin A6 OS=Homo sapiens GN=ANXA6 PE=1 SV=3	P08133	1.0%	98.8%	26	4046	673.00	14.205447	2

23	61364.7	1	>sp|Q9H223|EHD4_HUMAN EH domain-containing protein 4 OS=Homo sapiens GN=EHD4 PE=1 SV=1	Q9H223	0.9%	94.8%	18	3012	541.00	13.155331	1
21	103563	1	>sp|P12814|ACTN1_HUMAN Alpha-actinin-1 OS=Homo sapiens GN=ACTN1 PE=1 SV=2	P12814	0.9%	92.5%	16	4811	892.00	12.744259	1
8	262638	4	>tr|A0A024R462|A0A024R462_HUMAN Fibronectin 1, isoform CRA_n OS=Homo sapiens GN=FN1 PE=4	A0A024R462	0.8%	99.0%	52	11984	2355.00	12.024166	4
20	70881.2	1	>sp|Q13492|PICAL_HUMAN Phosphatidylinositol-binding clathrin assembly protein OS=Homo sapiens GN=PICALM PE=1 SV=2	Q13492	0.7%	82.7%	12	2953	652.00	10.701876	1
13	280154	2	>sp|O75369|FLNB_HUMAN Filamin-B OS=Homo sapiens GN=FLNB PE=1 SV=2	O75369	0.2%	96.9%	21	2534	2602.00	2.3011419	2
25	124292	1	>sp|P18206|VINC_HUMAN Vinculin OS=Homo sapiens GN=VCL PE=1	P18206	0.1%	87.3%	13	839	1134.00	1.7482075	1

CHENZIQI_S2_032022

Hits	Protein M:	No. of	Sequence Header	Link	Relativ	Probabi	Score	SI	AA No	SIn*1E6	No. of Unique Peptide
2	53676.2	22	>sp|P08670|VIME_HUMAN Vimentin OS=Homo sapiens GN=VIM PE=1	P08670	21.7%	99.0%	324	3E+05	466.00	429.0539	16
1	71244.3	26	>sp|P02769|ALBU_BOVIN Serum albumin OS=Bos taurus GN=ALB PE=1 SV=4	P02769	15.7%	99.0%	339	3E+05	607.00	310.58607	16
3	46525.3	18	>sp|P50454|SERPH_HUMAN Serpin H1 OS=Homo sapiens GN=SERPINH1 PE=1 SV=2	P50454	11.4%	99.0%	240	1E+05	418.00	224.02695	13
13	48283	5	>sp|Q8NBS9|TXND5_HUMAN Thioredoxin domain-containing protein 5 OS=Homo sapiens GN=TXNDC5 PE=1 SV=2	Q8NBS9	6.6%	99.0%	70	75445	432.00	129.98015	5
5	42051.9	12	>sp|P60709|ACTB_HUMAN Actin, cytoplasmic 1 OS=Homo sapiens GN=ACTB PE=1 SV=1	P60709	5.9%	99.0%	159	58292	375.00	115.69325	7
36	46441.1	1	>sp|Q14032|BAAT_HUMAN Bile acid-CoA:amino acid N-acyltransferase OS=Homo sapiens GN=BAAT PE=1 SV=1	Q14032	4.8%	85.5%	13	52696	418.00	93.827835	1
4	59908.4	14	>tr|L8B4I8|L8B4I8_HUMAN Ubiquitin C OS=Homo sapiens GN=UbC PE=4	L8B4I8	3.2%	98.4%	182	44688	533.00	62.401357	2
10	59827.7	8	>sp|P25705|ATPA_HUMAN ATP synthase subunit alpha, mitochondrial OS=Homo sapiens GN=ATP5A1 PE=1 SV=1	P25705	3.1%	99.0%	129	45210	553.00	60.847075	7
9	83554.3	9	>sp|P08238|HS90B_HUMAN Heat shock protein HSP 90-beta OS=Homo sapiens GN=HSP90AB1 PE=1 SV=4	P08238	2.5%	99.0%	96	47845	724.00	49.184507	5
11	56524.7	7	>sp|P06576|ATPB_HUMAN ATP synthase subunit beta, mitochondrial OS=Homo sapiens GN=ATP5B PE=1 SV=3	P06576	2.4%	99.0%	104	33085	529.00	46.548511	7
7	45088.1	10	>sp|P05121|PAI1_HUMAN Plasminogen activator inhibitor 1 OS=Homo sapiens GN=SERPINE1	P05121	2.3%	99.0%	123	24089	402.00	44.598787	6
22	50495.3	3	>sp|Q5VTE0|EF1A3_HUMAN Putative elongation factor 1-alpha-like 3 OS=Homo sapiens GN=EEF1A1P5 PE=5 SV=1	Q5VTE0	2.1%	99.0%	51	26275	462.00	42.328324	3
14	50787.9	4	>sp|Q71U36|TBA1A_HUMAN Tubulin alpha-1A chain OS=Homo sapiens GN=TUBA1A PE=1 SV=1	Q71U36	1.9%	99.0%	58	23132	451.00	38.173939	4
8	74379.9	9	>sp|P02545|LMNA_HUMAN Prelamin-A/C OS=Homo sapiens GN=LMNA PE=1 SV=1	P02545	1.7%	99.0%	132	30741	664.00	34.45722	9
12	47481.5	6	>sp|P06733|ENOA_HUMAN Alpha-enolase OS=Homo sapiens GN=ENO1 PE=1 SV=2	P06733	1.5%	99.0%	68	16858	434.00	28.909904	6

17	50095.2	3	>sp|P07437|TBB5_HUMAN Tubulin beta chain OS=Homo sapiens GN=TUBB PE=1 SV=2	P07437	1.4%	99.0%	40	16899	444.00	28.327508	3
18	46352.7	3	>sp|P60842|IF4A1_HUMAN Eukaryotic initiation factor 4A-I OS=Homo sapiens GN=EIF4A1 PE=1 SV=1	P60842	1.4%	99.0%	49	15022	406.00	27.537978	3
30	49483.5	2	>sp|P31943|HNRH1_HUMAN Heterogeneous nuclear ribonucleoprotein H OS=Homo sapiens GN=HNRNPH1 PE=1 SV=4	P31943	1.3%	99.0%	38	14908	449.00	24.711743	2
19	50429.3	3	>sp|P26641|EF1G_HUMAN Elongation factor 1-gamma OS=Homo sapiens GN=EEF1G PE=1 SV=3	P26641	1.2%	99.0%	37	13487	437.00	22.970172	3
24	37178.4	2	>tr|F8W914|F8W914_HUMAN Reticulon OS=Homo sapiens GN=RTN4 PE=1 SV=1	F8W914	1.0%	99.0%	30	9460	345.00	20.40809	2
15	48490.4	3	>sp|Q15084|PDIA6_HUMAN Protein disulfide-isomerase A6 OS=Homo sapiens GN=PDIA6 PE=1 SV=1	Q15084	1.0%	99.0%	55	11758	440.00	19.888916	3
6	227645	11	>sp|P35579|MYH9_HUMAN Myosin-9 OS=Homo sapiens GN=MYH9 PE=1 SV=4	P35579	0.9%	99.0%	184	47238	1960.00	17.937659	11
16	51087.1	3	>sp|P50395|GDIB_HUMAN Rab GDP dissociation inhibitor beta OS=Homo sapiens GN=GDI2 PE=1 SV=2	P50395	0.6%	99.0%	40	7482	445.00	12.513766	3
21	50940.1	3	>sp|P39656|OST48_HUMAN Dolichyl-diphosphooligosaccharide--protein glycosyltransferase 48 kDa subunit OS=Homo sapiens GN=DDOST PE=1 SV=4	P39656	0.6%	99.0%	45	7117	456.00	11.616156	3
31	49611.2	2	>sp|O00148|DX39A_HUMAN ATP-dependent RNA helicase DDX39A OS=Homo sapiens GN=DDX39A PE=1 SV=2	O00148	0.5%	98.0%	24	5366	427.00	9.3530471	2
39	38807.9	1	>sp|P07355|ANXA2_HUMAN Annexin A2 OS=Homo sapiens GN=ANXA2 PE=1 SV=2	P07355	0.4%	86.9%	13	3571	339.00	7.8400797	1
32	80344.9	1	>sp|Q12931|TRAP1_HUMAN Heat shock protein 75 kDa, mitochondrial OS=Homo sapiens GN=TRAP1 PE=1	Q12931	0.4%	94.5%	18	7023	704.00	7.4247244	1
35	66096.9	1	>sp|Q07065|CKAP4_HUMAN Cytoskeleton-associated protein 4 OS=Homo sapiens GN=CKAP4 PE=1 SV=2	Q07065	0.3%	87.9%	14	5487	602.00	6.7837338	1
33	49228.3	1	>sp|P62495|ERF1_HUMAN Eukaryotic peptide chain release factor subunit 1 OS=Homo sapiens GN=ETF1 PE=1 SV=3	P62495	0.3%	85.3%	12	3796	437.00	6.4650977	1
28	72402.5	2	>sp|P11021|GRP78_HUMAN 78 kDa glucose-regulated protein OS=Homo sapiens GN=HSPA5 PE=1 SV=2	P11021	0.3%	99.0%	32	5575	654.00	6.3445007	2
27	103563	2	>sp|P12814|ACTN1_HUMAN Alpha-actinin-1 OS=Homo sapiens GN=ACTN1 PE=1 SV=2	P12814	0.3%	99.0%	32	6824	892.00	5.6938299	2
29	71082.4	2	>sp|P11142|HSP7C_HUMAN Heat shock cognate 71 kDa protein OS=Homo sapiens GN=HSPA8 PE=1	P11142	0.3%	99.0%	30	4929	646.00	5.6788007	2
23	47217	2	>sp|O60664|PLIN3_HUMAN Perilipin-3 OS=Homo sapiens GN=PLIN3 PE=1 SV=3	O60664	0.3%	98.6%	28	3176	434.00	5.446545	2
26	118051	2	>sp|Q8IVF7|FMNL3_HUMAN Formin-like protein 3 OS=Homo sapiens GN=FMNL3 PE=1 SV=3	Q8IVF7	0.3%	78.9%	20	7453	1028.00	5.3959552	1
25	133291	2	>sp|P07996|TSP1_HUMAN Thrombospondin-1 OS=Homo sapiens GN=THBS1 PE=1 SV=2	P07996	0.1%	98.4%	26	4638	1170.00	2.9503614	2
34	44414.2	1	>sp|Q15427|SF3B4_HUMAN Splicing factor 3B subunit 4 OS=Homo sapiens GN=SF3B4 PE=1 SV=1	Q15427	0.1%	82.7%	12	1477	424.00	2.5926564	1
20	262638	3	>tr|A0A024R462|A0A024R462_HUMAN Fibronectin 1, isoform CRA_n OS=Homo sapiens GN=FN1 PE=4	A0A024R462	0.1%	99.0%	42	8139	2355.00	2.5722339	3

37	280154	1	>sp|O75369|FLNB_HUMAN Filamin-B OS=Homo sapiens GN=FLNB PE=1 SV=2	O75369	0.1%	87.1%	13	3906	2602.00	1.1172626	1
38	229826	1	>sp|P35580|MYH10_HUMAN Myosin-10 OS=Homo sapiens GN=MYH10 PE=1 SV=3	P35580	0.1%	90.4%	16	2752	1976.00	1.0365537	1
